# A *Drosophila* chemical screen reveals synergistic effect of MEK and DGKα inhibition in Ras-driven cancer

**DOI:** 10.1242/dmm.049769

**Published:** 2023-04-03

**Authors:** John E. La Marca, Robert W. Ely, Sarah T. Diepstraten, Peter Burke, Gemma L. Kelly, Patrick O. Humbert, Helena E. Richardson

**Affiliations:** ^1^ Department of Biochemistry & Chemistry, La Trobe Institute for Molecular Science, La Trobe University, Bundoora, Victoria 3086, Australia; ^2^ Blood Cells and Blood Cancer Division, Water and Eliza Hall Institute, Melbourne, Victoria 3052, Australia; ^3^ Department of Medical Biology, University of Melbourne, Melbourne, Victoria 3010, Australia; ^4^ Research Division, Peter MacCallum Cancer Centre, Melbourne, Victoria 3002, Australia; ^5^ Sir Peter MacCallum Department of Oncology, Department of Biochemistry & Pharmacology, and Department of Clinical Pathology, University of Melbourne, Melbourne, Victoria 3010, Australia; ^6^ Sir Peter MacCallum Department of Oncology, Department of Biochemistry & Pharmacology, and Department of Anatomy & Neuroscience, University of Melbourne, Melbourne, Victoria 3010, Australia

**Keywords:** *Drosophila*, *Ras*, *scrib*, Diacyl glycerol kinase α (DGKα), Chemical screening, Synergism

## Abstract

Elevated Ras signalling is highly prevalent in human cancer; however, targeting Ras-driven cancers with Ras pathway inhibitors often leads to undesirable side effects and to drug resistance. Thus, identifying compounds that synergise with Ras pathway inhibitors would enable lower doses of the Ras pathway inhibitors to be used and also decrease the acquisition of drug resistance. Here, in a specialised chemical screen using a *Drosophila* model of Ras-driven cancer, we have identified compounds that reduce tumour size by synergising with sub-therapeutic doses of the Ras pathway inhibitor trametinib, which targets MEK, the mitogen-activated protein kinase kinase, in this pathway. Analysis of one of the hits, ritanserin, and related compounds revealed that diacyl glycerol kinase α (DGKα, Dgk in *Drosophila*) was the critical target required for synergism with trametinib. Human epithelial cells harbouring the *H-RAS* oncogene and knockdown of the cell polarity gene *SCRIB* were also sensitive to treatment with trametinib and DGKα inhibitors. Mechanistically, DGKα inhibition synergises with trametinib by increasing the P38 stress-response signalling pathway in *H-RAS^G12V^ SCRIB^RNAi^* cells, which could lead to cell quiescence. Our results reveal that targeting Ras-driven human cancers with Ras pathway and DGKα inhibitors should be an effective combination drug therapy.

## Teaser

**Summary:** Using a *Drosophila* Ras-driven cancer model, inhibitors of diacyl glycerol kinase α (DGKα) were identified as being synergistic with low doses of the Ras pathway inhibitor, trametinib.

## INTRODUCTION

Cancer places a huge burden on public health outcomes worldwide, with estimated new cancer cases numbering almost 20 million annually, and associated deaths approximated at 10 million for the year 2020 (The Global Cancer Observatory, 2020). Coupled with these staggering rates of incidence are the difficulties involved in creating new and improved treatments for cancer. Emerging anti-cancer drugs have the lowest likelihood of moving forward from phase 1 trials compared to all other classes of drugs, and the list of new drug candidates gaining US Food and Drug Administration (FDA) approval for clinical use leaves much to be desired (Hay et al., 2014). Thus, the field of oncology could benefit from enhanced and more immediate methods for drug discovery, or the repurposing of drugs that have already been approved by the FDA. One model that can aid in this process is the vinegar fly, *Drosophila melanogaster*, which can be used to screen for novel anti-cancer compounds *in vivo*.

*Drosophila* is used extensively in the study of many genetic diseases (Bier, 2005; Wangler et al., 2015, 2017) and has been used for the investigation of cancer for over 100 years (Gonzalez, 2013; Stark, 1918). *Drosophila* carries orthologues of 68% of known human cancer-causing genes, and there is also a high level of conservation between human and *Drosophila* biological processes and signalling pathways (Brumby and Richardson, 2005; Hanahan and Weinberg, 2011). These factors, as well as its short life cycle and low maintenance costs, position *Drosophila* as an invaluable tool, filling a niche between mammalian cell lines and more complex organisms for the study of cancer both *in vitro* and *in vivo,* and also enables its use as a platform for the identification of anti-cancer compounds (Bangi, 2019; Brumby and Richardson, 2005; Gladstone and Su, 2011; Gonzalez, 2013; Richardson et al., 2015; Rudrapatna et al., 2012). Importantly, a majority of the hallmarks of cancer can be modelled in *Drosophila*, including increased cell proliferation, evasion of apoptosis and differentiation, and induction of invasion/metastasis (Brumby and Richardson, 2005; Hanahan and Weinberg, 2011).

A group of genes heavily implicated in human cancers are the oncogenic RAS genes, which signal through the RAF-MEK-MAPK and phospho-inositol-3-kinase (PI3K)-AKT-mechanistic target of rapamycin (mTOR) pathways to drive cell growth, proliferation and survival (Malumbres and Barbacid, 2003; Pylayeva-Gupta et al., 2011). However, oncogenic mutations in RAS genes are not sufficient to drive malignant cancers as high levels of Ras signalling lead to a cell cycle arrest and senescence and, therefore, additional mutations are needed to overcome these curbs to cancer progression (Coleman et al., 2006; DeNicola and Tuveson, 2009; Dimauro and David, 2010; Olson et al., 1998; Sahai et al., 2001). The *Drosophila* orthologue of the human RAS genes is *Ras85D* (hereafter termed *Ras*). Expression of an activated form of *Drosophila Ras*, termed *Ras^V12^* (which bears the constitutively activating G12V mutation, a frequent mutation in human RAS orthologues), induces hyperplastic growth in a variety of tissues, but further tumour progression does not occur due to cell cycle arrest, differentiation and senescence-like characteristics (Brumby et al., 2011; Ito and Igaki, 2021; Karim and Rubin, 1998; Nakamura and Igaki, 2017).

A major factor in tumourigenesis is the loss of cell polarity, with the majority of human epithelial tumours estimated to have cell polarity and tissue architecture disruption (Gödde et al., 2014; Lee and Vasioukhin, 2008; Muthuswamy and Xue, 2012; Royer and Lu, 2011). Indeed, the disruption of cell polarity is implicated as a causative factor in many different cancers [such as breast and cervical cancers (Feigin et al., 2014; Thomas et al., 2008)], with cell polarity genes acting as so-called ‘tumour suppressors’ (Bilder, 2004; Sonoshita and Cagan, 2017). The *Drosophila* genes *lethal (2) giant larvae* [*l(2)gl*], *discs large 1* (*dlg1*) and *scribble* (*scrib*) are cell polarity genes that, when mutated, result in cells exhibiting a loss of polarity and tissue architecture, disrupted differentiation and increased tissue growth. Upon transplantation into adult flies, these mutant cells massively overgrow and undergo invasion/metastasis reminiscent of mammalian cancers (Froldi et al., 2008). Mammalian orthologs of *l(2)gl*, *Dlg1* and *scrib* similarly act as tumour suppressors, restraining cell proliferation and invasion/metastasis (Elsum et al., 2012; Humbert et al., 2008; Stephens et al., 2018).

When *Drosophila* cell polarity genes are mutated, the tissue exhibits overgrowth through impairment of the Hippo pathway, a negative tissue growth control pathway; its downstream target Yorkie (Yki) functions as a co-transcriptional activator to drive expression of the cell growth/proliferation genes *myc* and *Cyclin E* (*CycE*), and the anti-apoptotic gene *Death-associated inhibitor of apoptosis 1* (*Diap1*), thereby causing increased cell proliferation and survival (Doggett et al., 2011). When disrupted in clones within a whole tissue, mutant *scrib* cells do not display the phenotype of aggressive tumours and are largely eliminated via c-Jun N-terminal kinase (JNK)-mediated apoptosis (Brumby and Richardson, 2003; Leong et al., 2009). However, when *Ras^V12^* is expressed within epithelial tissues that are also mutant for *scrib*, neoplastic tumours are generated, which show increased cell proliferation, increased survival, reduced differentiation and invasive/metastatic behaviour, thereby replicating many of the mammalian cancer hallmarks (Brumby and Richardson, 2003; Leong et al., 2009; Pagliarini and Xu, 2003). Alone, *Ras^V12^* drives cell proliferation and survival, but also induces cell cycle arrest, senescence and differentiation (Brumby et al., 2011; Ito and Igaki, 2021; Karim and Rubin, 1998; Nakamura and Igaki, 2017). Aggressive and neoplastic properties arise through the cooperative effects of these two mutations, largely through combination of the consequences of tissue overgrowth and suppression of senescence by impaired Hippo pathway signalling, the pro-survival characteristics induced by Ras activation, and the hijacking of activated JNK signalling to block differentiation and induce invasion through the upregulation of matrix metalloproteases (Doggett et al., 2011; Igaki et al., 2006; Ito and Igaki, 2021; Leong et al., 2009; Uhlirova and Bohmann, 2006).

The simplified genetics of the fly (with fewer redundant genes than in mammals allowing for easier knockdown models), together with the ability to rear hundreds of animals that can easily have their diet supplemented with drugs and monitored in a whole-body environment, are powerful benefits to using *Drosophila* in a drug-discovery setting (Bangi, 2019; Gladstone and Su, 2011; Richardson et al., 2015; Yadav et al., 2016). An analysis of drugs specifically targeting various signalling pathways in *Drosophila* revealed that the mode of action of most pathways was conserved between flies and humans (Bangi et al., 2011), reinforcing the utility of *Drosophila* as a platform for drug discovery that is relevant to human biology. Indeed, several studies have utilised *Drosophila* as a model system for anti-cancer drug discovery, using a variety of approaches (Bangi et al., 2019, 2016; Dar et al., 2012; Edwards et al., 2011; Gladstone et al., 2012; Jaklevic et al., 2006; Levine and Cagan, 2016; Markstein et al., 2014; Sonoshita et al., 2018; Vidal et al., 2005).

In our previous study, we used a clonally induced, polarity-impaired Ras-driven (*scrib^−^/Ras^V12^*) model of cancer to screen a library containing 2000 compounds and identify drugs that reduced tumour size in *Drosophila* larvae (Willoughby et al., 2013). We developed a screening platform in a 96-well plate format, in which larvae were fed food containing different compounds for 5 days before being imaged to assess the effect on GFP-marked tumour size (Willoughby et al., 2013). In this screen, we identified acivicin, a glutamine analogue already known to have anti-tumour properties in humans, and, upon pharmacogenetic analysis, demonstrated that it targeted the glutamine-utilisation enzyme CTP synthase, as well as the tricarboxylic acid cycle (Willoughby et al., 2013).

In the current study, we adapted our *scrib^−^/Ras^V12^* model and the larval screening platform we developed to screen for compounds that synergistically inhibit tumour growth together with the mitogen-activated protein kinase kinase (MEK) inhibitor trametinib. Trametinib is an orally administered, FDA-approved, widely used drug for the treatment of Ras-driven cancers, including melanoma, non-small-cell lung cancer, thyroid cancer and glioma (Ferrari et al., 2020; Hoffner and Benchich, 2018; Manoharan et al., 2020; Tabbò et al., 2022; Wright and McCormack, 2013; Zeiser, 2014). However, trametinib has dermatological side effects that often lead to the dose being lowered or treatment withdrawn (Abdel-Rahman et al., 2015; Anforth et al., 2014). Thus, identifying compounds that can effectively synergise with trametinib to inhibit Ras pathway-driven cancers, enabling lower doses of trametinib to be used, would be highly desirable. After screening ∼5000 compounds, we identified two compounds that synergised with a sub-therapeutic dose of trametinib to reduce tumour size: the Polo-like kinase inhibitor, volasertib, and the serotonin receptor and diacyl glycerol kinase (DGK) inhibitor, ritanserin. Pharmacogenetic analyses of the mode of action of ritanserin indicated that inhibition of DGK is required to reduce tumour size in cooperation with trametinib. We demonstrate that this synergistic mechanism between trametinib and DGK inhibitors is conserved in human mammary epithelial cells and reveal that the drug combination leads to upregulation of the stress-response P38 pathway in *SCRIB*-knockdown *H-RAS^G12V^*-expressing cells. Thus, low-dose trametinib combined with ritanserin, or with more selective DGKα inhibitors, is a novel anti-cancer combination therapy that could be developed to target human mammary cancers, as well as other human Ras-driven polarity-impaired cancers.

## RESULTS

### Identification of compounds that synergise with a sub-therapeutic dose of the MEK inhibitor trametinib to suppress polarity-impaired Ras-driven tumour growth in *Drosophila*

**Fig. 1.**
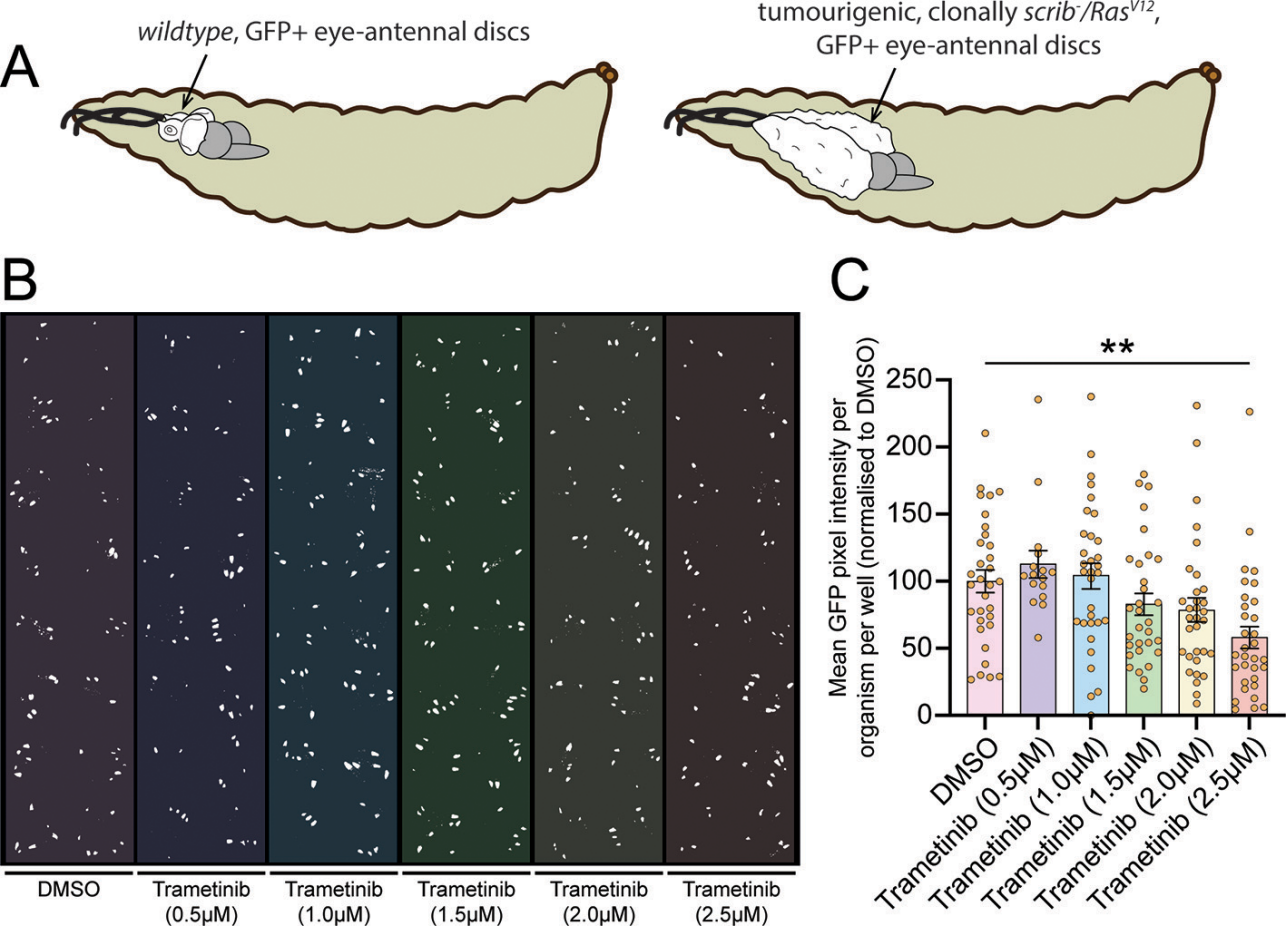
**Titration of trametinib fed to *scrib^−^/Ras^V12^* tumour-bearing larvae reveals the minimum concentration needed for tumour growth inhibition.** (A) Diagram of the model system in the eye-antennal discs. Larvae with wild-type, GFP-marked eye-antennal discs develop normally. In larvae with GFP-marked, *scrib^−^*/*Ras^V12^* clones in their eye-antennal discs, the tissue substantially overgrows: changes that are visualisable/quantifiable via fluorescence microscopy. (B) Binarised image of a drug test plate where larvae were treated with different concentrations of the MEK inhibitor trametinib on larvae possessing *scrib^−^/Ras^V12^* tumours, with the GFP-marked tumours visualised in white (see Materials and Methods for details). (C) Quantifications of GFP pixel intensity revealed that, at a final concentration of 1.5 µM and above, trametinib reduced the tumour size in the larvae, with 2.5 µM trametinib achieving knockdown to ∼60% of that of the DMSO-treated control. Results represent two replicate experiments with 16 (for trametinib at 0.5 µM) or 32 (for other treatments) replicate wells for each treatment. Statistical test used was a one-way ANOVA with Tukey's multiple comparisons, with non-significant comparisons not visualised. Error bars represent s.e.m. **P<0.01.

Using the clonally induced *scrib* mutant, oncogenic *Ras* (*Ras^V12^*) model of epithelial tumourigenesis (Brumby and Richardson, 2003; Leong et al., 2009), we have previously shown that feeding tumour-bearing larvae a bioavailable compound (PD0325901) that targets MEK (*Drosophila* Dsor1), a protein kinase in the Ras-MAPK pathway, was effective in reducing tumour size (Willoughby et al., 2013). As another MEK inhibitor, trametinib, is a widely used, orally administered drug for the treatment of Ras-driven human cancers (Ferrari et al., 2020; Hoffner and Benchich, 2018; Manoharan et al., 2020; Tabbò et al., 2022; Wright and McCormack, 2013; Zeiser, 2014), we tested whether it was also effective in reducing tumour size of *scrib* mutant, *Ras^V12^*-expressing (*scrib^−^*/*Ras^V12^*) larvae when administered in the food, as measured by changes in the size of the GFP-marked eye-antennal discs, which overgrow substantially upon induction of *scrib^−^*/*Ras^V12^* clone generation (Fig. 1A). Indeed, we found that trametinib at 5-10 µM was highly effective in reducing the tumour size in these tumour-bearing larvae, and, upon titration, we found that a final concentration of 2.5 µM trametinib generally resulted in a 50-70% average reduction of tumour size (Fig. 1B,C). Thus, we hypothesised that we would be able to identify compounds that could synergise with trametinib in inhibiting tumour size by using a dose of 2.5 µM trametinib (which we define as sub-therapeutic) in a chemical screen.

**Fig. 2.**
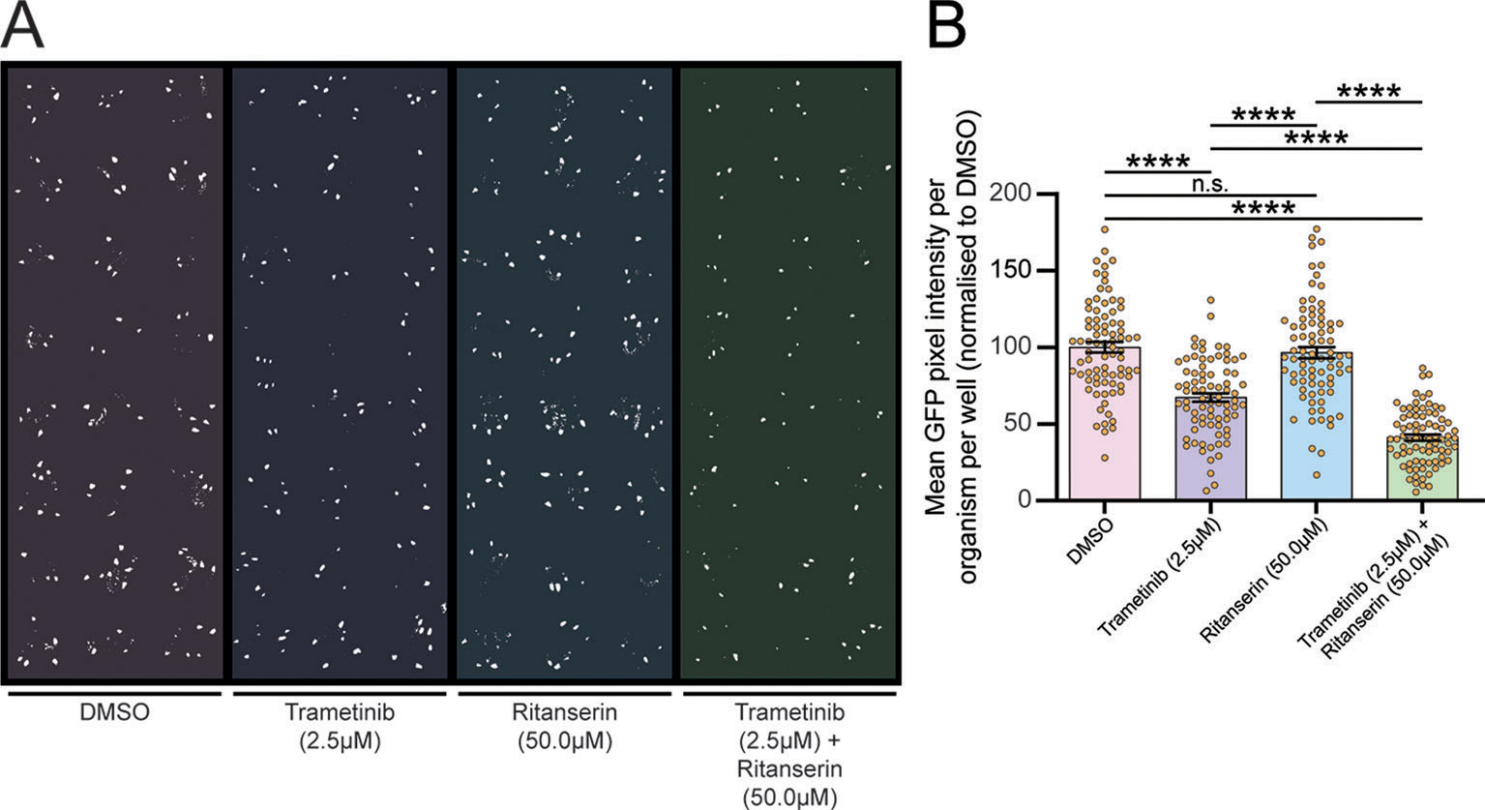
**Ritanserin shows synergy with trametinib in reducing the tumour size in *scrib^−^/Ras^V12^* tumour-bearing larvae.** (A) Binarised image of a drug test plate where larvae were treated with trametinib in combination with the serotonin receptor and diacyl glycerol kinase inhibitor, ritanserin. (B) Quantifications of GFP pixel intensity revealed that ritanserin synergised with trametinib (at final concentrations of 50 µM ritanserin and 2.5 µM trametinib) to significantly reduce tumour size to ∼40% of that of the DMSO-treated control. Data are from four replicate experiments with 16-24 replicate wells for each treatment. Statistical test used was a one-way ANOVA with Tukey's multiple comparisons. Error bars represent s.e.m. n.s., not significant; ****P<0.0001.

To identify compounds that could synergise with the Ras-MAPK pathway inhibitor trametinib, we screened four specialised compound libraries (obtained from the Walter and Eliza Hall Institute): 276 kinase inhibitors; 89 epigenetic modifiers; 179 targeted compounds; and 3707 known drugs (File S1). We assessed their effectiveness at reducing *scrib^−^/Ras^V12^* tumour size combined with a sub-therapeutic dose of trametinib (2.5 µM), which only minimally affected the tumour size in isolation. We screened the tumour-bearing larvae in 96-well deep-well micro-titre plates to which different drugs were administered in the food at a final concentration of 50 µM, along with DMSO control wells, as previously described (Richardson et al., 2015; Willoughby et al., 2013). We screened the first three libraries in duplicate with or without sub-therapeutic levels of trametinib, but as the known drug library was in limited supply, we only screened one copy of this library in the presence of trametinib. From this screen, we identified 20 potential hits that reduced tumour size in all larvae in the well relative to the DMSO control wells for each plate, but which did not appear to reduce overall larval size. These compounds included trametinib itself, as well as compounds targeting a variety of other pathways (Table 1). To validate the candidates, we tested independently sourced supplies of these 19 novel compounds, with or without a sub-therapeutic dose of trametinib in multiple wells of the micro-titre plates. This analysis revealed that 13 of the compounds did not significantly reduce the size of *scrib^−^/Ras^V12^* tumours (File S2). Four compounds, methotrexate and pralatrexate (folate antagonists), temsirolimus (mTOR inhibitor), and GSK2126458 (PI3K inhibitor), were confirmed to reduce tumour size, but did not synergize with trametinib at the various doses tested (Figs S1 and S2), and therefore were not further analysed. Importantly, two compounds were confirmed to display synergism (defined by a greater than additive effect) with trametinib in reducing tumour size: volasertib (Polo-like kinase inhibitor) (Schöffski, 2009) and ritanserin [serotonin receptor 5-HT2A/2C and DGKα inhibitor (Boroda et al., 2017; Leysen et al., 1985)] (Fig. 2; Fig. S3). At two different doses, volasertib showed a reduction in tumour size on its own, but at the lower dose (12.5 µM), it showed synergism with trametinib (Fig. S3A,B). Ritanserin (50 µM) did not show any significant effect on tumour size on its own but synergised with trametinib to significantly reduce tumour size from ∼75% to 50% (Fig. 2A,B). However, at lower doses of trametinib (1.25 µM), ritanserin (50 µM) was unable to reduce tumour size (Fig. S4), indicating that at least 2.5 µM trametinib is required for a robust synergistic response with ritanserin. Thus, we have discovered two compounds, volasertib and ritanserin, that synergise with trametinib to reduce *scrib^−^/Ras^V12^* tumour size. As volasertib had an effect on its own and as this class of inhibitors has already been extensively used in cancer therapy (Gjertsen and Schöffski, 2014; Shakeel et al., 2021; Zhang et al., 2021), we focused our attention on ritanserin, which had no significant effect on tumour size on its own and has no history of use as an anti-cancer drug.

**Table 1.**
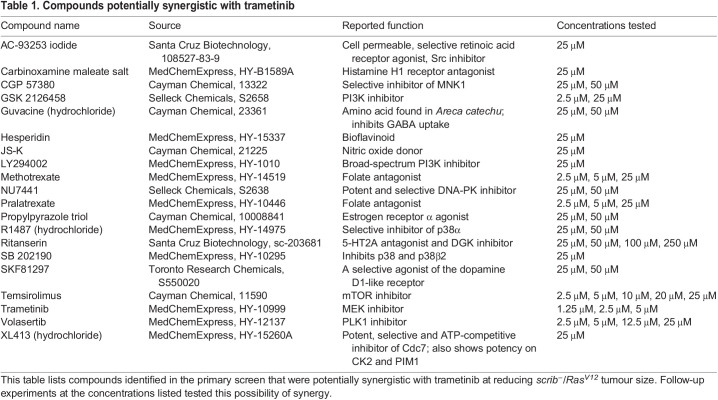
Compounds potentially synergistic with trametinib

### Ritanserin synergises with trametinib to target tumour growth and enable continued development of *scrib^−^/Ras^V12^* tumour-bearing *Drosophila* larvae

**Fig. 3.**
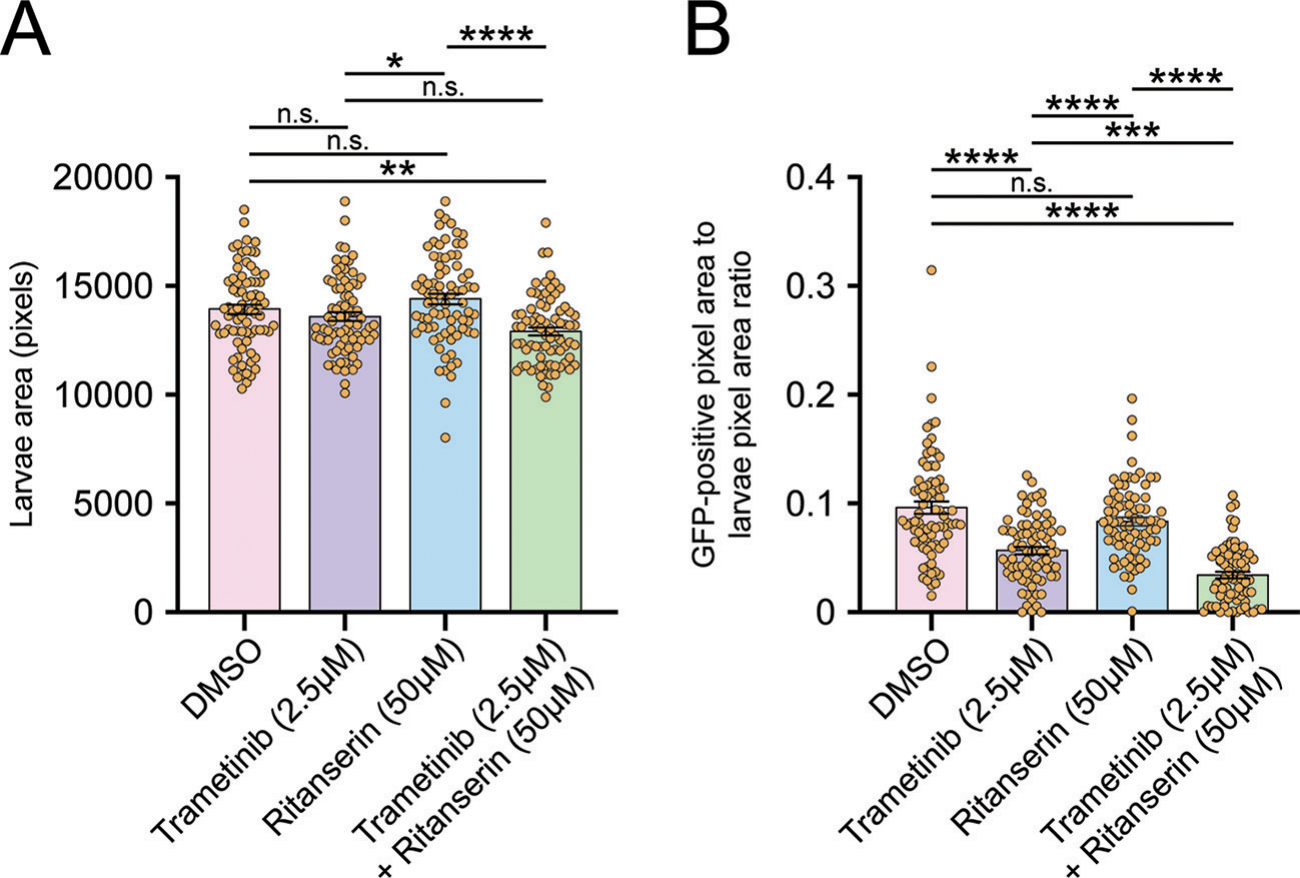
**Trametinib and ritanserin combination treatment directly targets *scrib^−^/Ras^V12^* tumour growth.** (A) Quantifications of larval size after treatment with trametinib and ritanserin revealed that there was a significant reduction in larval size in the larvae undergoing combination treatment compared to that of the DMSO controls, but there was no difference in larval size if larvae were treated with trametinib alone or with trametinib and ritanserin in combination. (B) Quantification of the ratio between GFP pixel intensity and larval area demonstrates that despite the reduction in larval size, the trametinib and ritanserin combination treatment led to a tumour size of ∼3.5% of the larvae area, a significant reduction compared to ∼9% in the DMSO control and ritanserin-alone treatments, and ∼5.5% in the trametinib-alone treatment. Data were derived from two replicate experiments with 16-23 replicate wells for each treatment. Statistical test used was a one-way ANOVA with Tukey's multiple comparisons. Error bars represent s.e.m. n.s., not significant; *P<0.05; **P<0.01; ***P<0.001; ****P<0.0001.

First, we sought to determine whether ritanserin with or without trametinib was specifically targeting the *scrib^−^/Ras^V12^* tumour, rather than affecting growth of the whole organism. To do this, we measured the size of whole larvae and their GFP-labelled tumours (*n*=78 per treatment) in drug-treated or DMSO vehicle-treated control samples (Fig. 3). We found that ritanserin treatment alone resulted in a small increase in the average larval size (Fig. 3A). By contrast, the low dose of trametinib did not affect *scrib^−^/Ras^V12^* tumour-bearing larval size, whereas the combined treatment of trametinib with ritanserin resulted in a small decrease in the average larval size (Fig. 3A). However, when the effects of the drugs on GFP-marked tumour sizes were presented as a ratio compared with whole larval sizes, ritanserin alone had no effect, trametinib reduced the tumour to ∼60% of the DMSO control, and the combined drug combination showed a reduction of the relative tumour size to ∼35% of the DMSO control (Fig. 3B). This result indicates that the ritanserin and trametinib drug combination targets the tumour specifically.

We next examined whether ritanserin with or without trametinib drug treatment could rescue development of the *scrib^−^/Ras^V12^* tumour-bearing larvae, which generally are overgrown owing to a block or delay in progressing to the pupal stage. Analysis of the number of pupae observed treated with DMSO, trametinib alone, ritanserin alone or with both drugs revealed ∼2-fold higher pupation rates with the combination treatment compared with single-drug treatments (Fig. S5). Thus, the ritanserin and trametinib drug combination increases the survival of tumour-bearing larvae and increases their ability to progress into the pupal stage, which is consistent with the effect of the drug combination in reducing tumour size.

### Ritanserin targets Dgk to synergise with trametinib in inhibiting Ras-driven polarity-impaired tumour growth in *Drosophila*

As ritanserin inhibits both DGKα and serotonin receptors (Boroda et al., 2017; Franks et al., 2017; Mizutani et al., 2018), we wished to determine whether Dgk or serotonin receptors are the key target for ritanserin in *Drosophila* in reducing *scrib^−^/Ras^V12^* tumour size in cooperation with trametinib by analysing specific DGKα or serotonin receptor inhibitors. Firstly, to confirm the results obtained with ritanserin, we analysed another DGKα/serotonin receptor inhibitory drug, R-59-022 (Boroda et al., 2017; Sato et al., 2013). Treatment of tumour-bearing larvae with R-59-022 together with a low dose of trametinib resulted in a significant reduction in tumour size (Fig. S6A,B) but had no significant effect alone, thereby complementing the results with ritanserin. We then analysed the DGKα specific inhibitors, Amb639752 (Velnati et al., 2019) and CU-3 (Liu et al., 2016; Yamaki et al., 2019). When treated with the DGKα-specific inhibitors Amb639752 or CU-3, together with a low dose of trametinib, significant decreases in tumour size were also observed (Fig. S6C-F), indicating that Dgk is the relevant target of ritanserin cooperating with trametinib in inhibiting Ras-driven polarity-impaired tumour growth. Consistent with these findings, treatment of the tumour-bearing larvae with specific serotonin receptor inhibitors, such as volinanserin (which has high selectivity for the 5-HT2A serotonin receptor) or paliperidone (which has highest affinity for the 5-HT2A and 5-HT7 serotonin receptors, but can also bind to α-adrenergic receptors) (Chue and Chue, 2012; Ebdrup et al., 2011; Jones et al., 2020), did not inhibit tumour growth alone or in combination with trametinib (Fig. S7A-D). In support of this, another specific serotonin receptor inhibitor, ketanserin, targeting the 5-HT2 family (Creed-Carson et al., 2011; Hedner and Persson, 1988), was tested in the primary screen and was not identified as being able to synergise with trametinib to reduce tumour size (File S1). Furthermore, multiple other serotonin receptor antagonists targeting 5-HT1, 5-HT2, 5-HT3, 5-HT4, 5-HT5, 5-HT6 and 5-HT7 families or multiple serotonin receptor types [e.g. methiothepin mesylate, 3-tropanylindole-3-carboxylate methiodide, 1-(1-naphthyl)piperazine hydrochloride, (S)-propranolol hydrochloride, S(−)-UH-301 hydrochloride, SB 200646 hydrochloride, cyclobenzaprine hydrochloride, 3-tropanyl-indole-3-carboxylate hydrochloride, SB 206553 hydrochloride, metoclopramide hydrochloride, SDZ-205,557 hydrochloride, granisetron hydrochloride, Ro 04-6790 dihydrochloride, SB 269970 hydrochloride, LY-310,762 hydrochloride, 5-carboxamidotryptamine maleate and methiothepin maleate] were tested in the primary screen but did not reduce tumour size (File S1). Although we cannot rule out the possibility that these compounds are simply not effective in *Drosophila*, these results overall indicate that the key target for ritanserin is Dgk, rather than serotonin receptors, in its synergistic effect with trametinib in reducing Ras-driven polarity-impaired tumour growth.

**Fig. 4.**
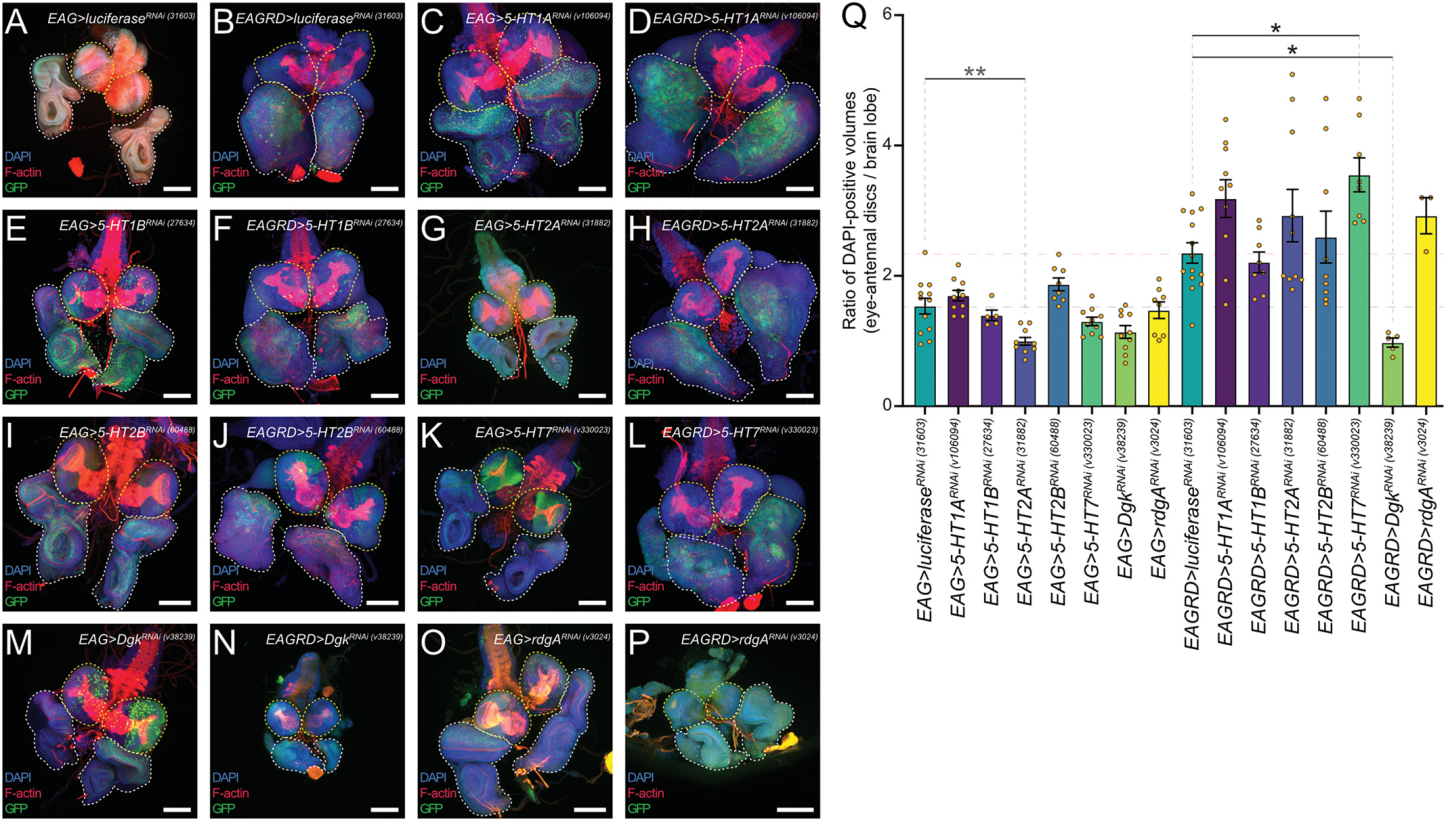
**Genetic analyses of *dlg1^RNAi^*/*Ras^V12^* tumours reveals that *Dgk* knockdown, but not *Dgkε, rdgA* or serotonin receptor gene knockdown, reduces tumour size.** (A-P) Brains from L3 animals with their attached wild-type (*EAG>*) (A,C,E,G,I,K,M,O) or *dlg1^RNAi^*/*Ras^V12^*-expressing (*EAGRD>*) (B,D,F,H,J,L,N,P) eye-antennal discs, also expressing GFP (green) and RNAi against 5HT (C-L) or DGK family genes (M-P), stained with DAPI (blue) and for F-actin (red). Brain lobes are outlined in yellow, and the eye-antennal discs are outlined in white. Scale bars: 100 µm. (Q) Quantification of the volume ratio between the eye-antennal discs and their respective brain lobes from samples in A-P. Little change was observed comparing the ratios of *EAG>* samples. However, in *EAGRD>* samples, *Dgk* knockdown significantly reduced the eye-antennal disc/brain lobe ratio, suggesting that *Dgk* is necessary for tumour growth. Statistical test used was a one-way ANOVA with Tukey's multiple comparisons, with non-significant comparisons not visualised. Error bars represent s.e.m. *P<0.05; **P<0.01.

To confirm these findings genetically, we used another *Drosophila* model of Ras-driven, polarity-impaired tumourigenesis, that of inducible knockdown of the *dlg1* polarity gene combined with expression of *Ras^V12^* within the whole eye-antennal tissue (Willecke et al., 2011). Using this model, we could induce tumours by outcrossing the stock to a neutral control (*UAS-luciferase*) or to *UAS-RNAi* lines targeting *Dgk* or serotonin receptor genes and, by comparison, assess the impact on tumour size. *Drosophila* has five serotonin receptor orthologs: 5-HT1A, 5-HT1B, 5-HT2A, 5-HT2B and 5-HT7 (Huser et al., 2017). Although *5-HT1B*, *5-HT2A* and *5-HT2B* mRNA expression are not detectable in the third instar larval stages (modENCODE RNAseq; *5-HT1B, 5-HT2A* and *5-HT2B*), we tested the effect of each gene individually using *UAS-RNAi* lines (which were confirmed as effective in knocking down the expression of their corresponding genes in wild-type adult/pupal tissue; Fig. S8) in the *dlg^RNAi^ /Ras^V12^* eye-antennal epithelial tumours and in wild-type eye epithelia (Fig. 4). Although knockdown of 5-HT2A had a small effect in reducing the size of otherwise wild-type eye discs (Fig. 4G, quantified in 4Q), the knockdown of these genes did not reduce tumour size and, in fact, knockdown of 5-HT7 slightly increased tumour size (Fig. 4D,F,H,J,L, quantified in 4Q). Although the knockdown efficiency of 5-HT2A and 5-HT7 using the RNAi lines was only at ∼40-50% when tested in wild-type tissues (Fig. S8), the fact that they have these phenotypic effects on tissue size suggests that their knockdown should have been effective enough to reduce tumour size if they were required for polarity-impaired Ras-driven tumour growth. Thus, these results did not provide any evidence that the serotonin receptor genes are individually required for polarity-impaired, Ras-driven tumourigenesis; however, we cannot rule out the possibility that stronger knockdown using different *UAS-RNAi* lines or simultaneous gene knockdowns may be required to reveal their role in tumour growth.

There are three DGK orthologs in *Drosophila*: *Dgk* (with the highest homology to mammalian *DGKA*, *DGKB* and *DGKG*), *Dgkε* (ortholog of mammalian *DGKE*) and *rdgA* (with highest homology to mammalian *DGKZ* and *DGKI*) (Mérida et al., 2008), which are all expressed in third instar larval tissue (modENCODE RNAseq; *Dgk*, *Dgkε* and *rdgA*). We tested the effect of knockdown of these genes on wild-type eye disc size and *dlg-RNAi Ras^V12^* tumour size (Fig. 4M-R, quantified in 4Q; Fig. S9) using RNAi lines that were shown to be highly effective at reducing mRNA levels (Fig. S8). Of these genes, only the knockdown of *Dgk* was able to significantly reduce tumour size (Fig. 4N, quantified in 4Q), but it had no effect on wild-type eye disc size (Fig. 4M, quantified in 4Q). Taken together with the results from the drug testing, these data provide evidence that *Dgk*, but not *Dgkε*, *rdgA* or the serotonin receptor genes, is required for Ras-driven polarity-impaired tumour growth.

### Chemical inhibition of DGKα reduces survival of *SCRIB*-knockdown *H-RAS^G12V^*-expressing human mammary epithelial cells

**Fig. 5.**
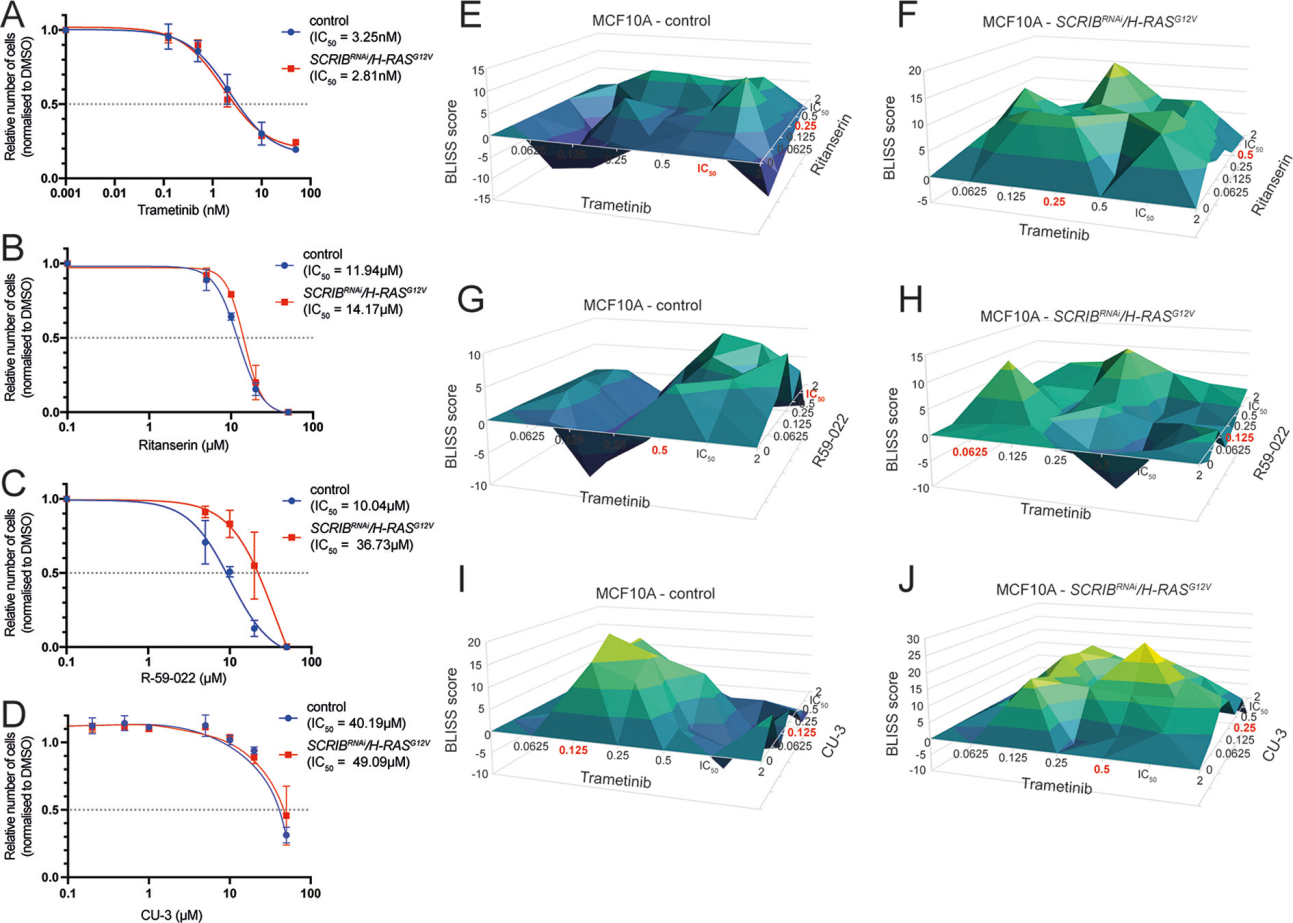
**Trametinib synergises with ritanserin, R-59-022 and CU-3 in cultured MCF10A cells expressing *SCRIB^RNAi^* and human *H-RAS^G12V^*.** (A-D) Dose response curves were generated for trametinib (A), ritanserin (B), R-59-022 (C) and CU-3 (D) treatments in MCF10A cells (both control cells and those with knockdown of *SCRIB* and expression of *H-RAS^G12V^*). Live-cell proportions were determined via CellTiter-Glo assay, and IC_50_ values were calculated from two to three independent experiments. (E-J) BLISS synergy scores for control (E,G,I) and *SCRIB^RNAi^*/*H-RAS^G12V^* (F,H,J) MCF10A cells treated with trametinib in combination with ritanserin (E,F), R-59-022 (G,H) or CU-3 (I,J) at the indicated doses, which are approximate magnitudes of the IC_50_ for each drug. The proportion of live cells was determined by CellTiter-Glo assay, across three replicate experiments. A BLISS score >0 indicates synergy. IC_50_ multiples for which highest synergy was achieved are indicated in red.

To determine whether our results in *Drosophila* were applicable in human cells, we used the normal mammary epithelial cell line, MCF10A, stably transformed with human *H-RAS^G12V^* (H-RAS bearing the constitutively activating G12V mutation) and *SCRIB^RNAi^* (Dow et al., 2008) (Fig. S10A), and tested whether ritanserin, R-59-022 or CU-3 were able to synergise with trametinib in reducing cell viability. We first conducted dose response analyses with these drugs to determine their effect on cell survival in MCF10A *SCRIB^RNAi^/H-RAS^G12V^* and control cells using the CellTiter-Glo assay, which measures metabolically active cells by quantifying the amount of ATP present, and calculated the half-maximal inhibitory concentration (IC_50_) for each drug (Fig. 5A-D). The MCF10A *SCRIB^RNAi^/H-RAS^G12V^* cells responded similarly to the control cells to each drug alone, except for R-59-022, for which the IC_50_ was ∼3.5 times higher for the *SCRIB^RNAi^/H-RAS^G12V^* cells (Fig. 5A-D). We then treated MCF10A *SCRIB^RNAi^/H-RAS^G12V^* and control cells with different dose combinations of trametinib and ritanserin, R-59-022 or CU-3 around the IC_50_ value for each drug, and measured cell survival (Fig. 5E-J; File S3). To determine synergy, we used the BLISS independence analysis (Goldoni and Tagliaferri, 2011), where a score >0 indicates synergy. For ritanserin and trametinib, although we found synergy between the drugs in both control cells (Fig. 5E) and *SCRIB^RNAi^/H-RAS^G12V^* cells (Fig. 5F), *SCRIB^RNAi^/H-RAS^G12V^* cells showed higher BLISS scores than control cells. The highest BLISS scores (∼15) in *SCRIB^RNAi^/H-RAS^G12V^* cells occurred at a quarter IC_50_ dose of trametinib and a half IC_50_ dose of ritanserin (Fig. 5F). Examining the effect of trametinib and ritanserin on MCF10A *H-RAS^G12V^* and MCF10A *SCRIB^RNAi^* cells revealed that the dose response to the drugs was similar between these cells (Fig. S10B,C), and that strong synergism between these drugs was observed (Fig. S10D,E). Overall, these results show that this drug combination could be used effectively to reduce the viability of both activated RAS and/or polarity-impaired cells. The combination of R-59-022 and trametinib (Fig. 5G,H) showed overall weak synergism in *SCRIB^RNAi^/H-RAS^G12V^* cells (BLISS scores of ∼10) and in control cells (BLISS scores <10). The DGKα-specific inhibitor, CU-3, showed strong synergy with trametinib in both control (Fig. 5I) and *SCRIB^RNAi^/H-RAS^G12V^* (Fig. 5J) cells, with the highest synergy scores (>25) obtained at less than half IC_50_ doses of each drug. To examine how the drugs impact cell viability, we performed cell cycle and death assays after 24 and 72 h of treatment with trametinib, ritanserin and CU-3, both individually and in combination. In these assays, we used the IC_50_ concentrations of each drug determined for control cells (Fig. 5A,B,D) in order to induce a strong phenotype. We saw a strong inhibition of the cell cycle in each cell line at the G1 phase, driven by the presence of trametinib (Fig. S11A,B and Fig. S12). By contrast, we did not observe a strong increase in cell death in any of the drug treatments of the cell lines at 24 or 72 h (Fig. S11C,D), although a small but significant decrease was observed upon trametinib and CU-3 combination drug treatment relative to DMSO in control and *scrib-RNAi* cells at 72 h. These results suggest that cell cycle arrest in G1 phase is largely responsible for the changes in cell viability observed in the CellTiter-Glo BLISS assays. Although no strong effect on cell death was observed upon trametinib and CU-3 combination drug treatment at 72 h, it is possible that this treatment may result in cell death at a later time or induce cell quiescence [G0 cell cycle arrest, resulting in a lower metabolic state (Marescal and Cheeseman, 2020)] to account for the decreased viability observed in the CellTiter-Glo assay. Together, these studies show that the synergistic interactions between trametinib and ritanserin, R-59-022 or CU-3 in inhibiting Ras-driven polarity-impaired tumour growth in *Drosophila* are translatable to human cells that have oncogenic RAS and polarity-impairment.

### Signalling pathways affected by the trametinib and CU-3 drug combination

**Fig. 6.**
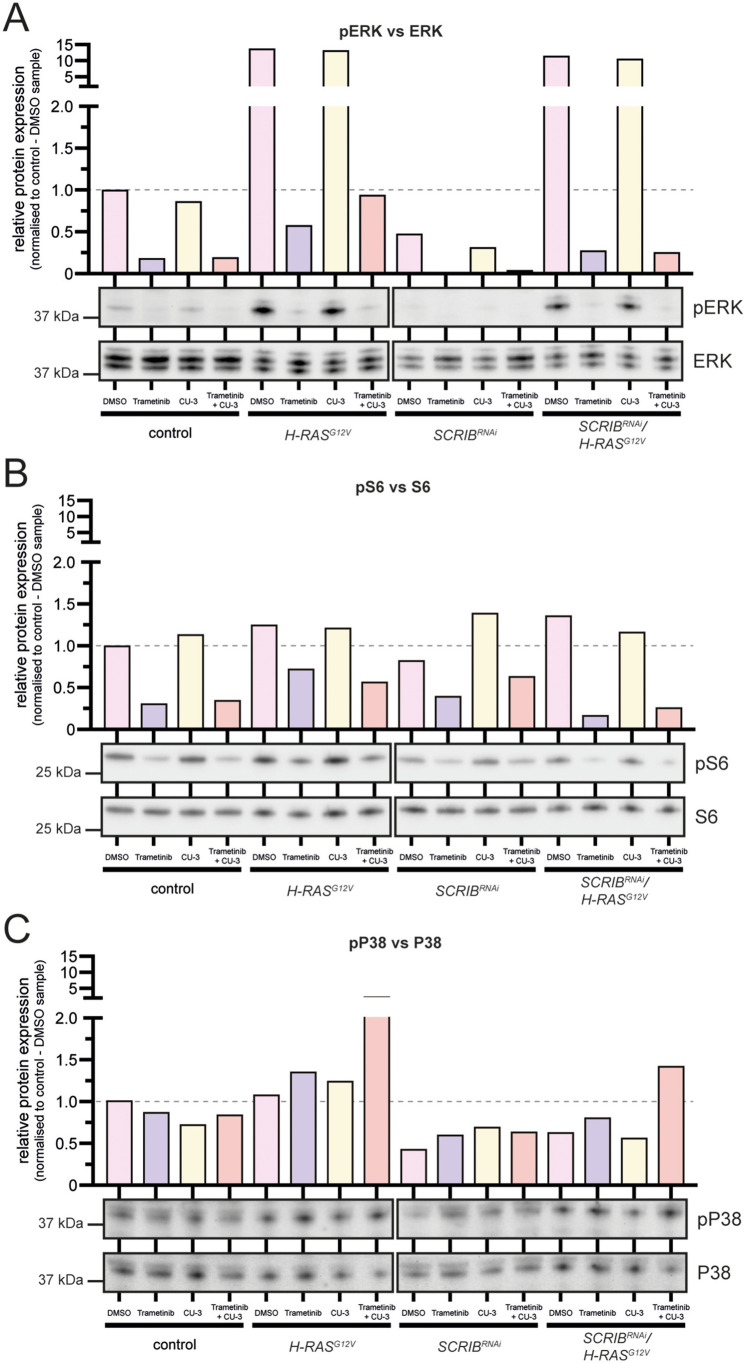
**The specific DGKα inhibitor CU-3 synergises with trametinib by activating the P38 signalling pathway in *H-RAS^G12V^*-expressing cells.** (A-E) Western blot images of MCF10A control, *H-RAS^G12V^*, *SCRIB^RNAi^* or *SCRIB^RNAi^/H-RAS^G12V^* cells, treated for 24 h with DMSO or the maximum synergy doses of trametinib, CU-3, or the combination of trametinib and CU-3. Quantifications are shown normalised to the MCF10A control line DMSO sample. Our data reveal that strong reductions in both pERK/total ERK (A) and pS6/total S6 (B) ratios are largely driven by the trametinib treatment, and that upregulation of the pP38/total P38 ratio (C) occurs in *H-RAS^G12V^*-expressing cells upon trametinib and CU-3 treatment, suggesting that activation of P38 may be responsible for the drug synergy.

As trametinib inhibits MEK in the Ras pathway and CU-3 inhibits DGKα (Liu et al., 2016; Yamaki et al., 2019), which phosphorylates diacyl glycerol (DAG) to generate phosphatidic acid (PA), an important secondary messenger lipid involved in the regulation of the Ras and mTOR signalling pathways (Andresen et al., 2002; Foster et al., 2014; Zhang and Du, 2009), we initially focused on how CU-3 with or without trametinib affected the Ras and mTOR pathways in the different cell lines. Accordingly, we treated control, *H-RAS^G12V^*, *SCRIB^RNAi^* and *SCRIB^RNAi^/H-RAS^G12V^* cells with the drugs individually or in combination, or with the DMSO vehicle control, for 24 h and analysed cell lysates for the levels of phosphorylated ERK (pERK; a MEK target), versus total ERK (Fig. 6A) and for the levels of phosphorylated S6 kinase (pS6; an mTOR target) versus total S6 kinase protein levels (Fig. 6B). For these experiments, we chose the concentrations of CU-3 (10 µM) and trametinib (1.515 nM) that showed the most effective synergistic effects in the control cells in the BLISS assay (Fig. 5J). As expected, we found that pERK levels were upregulated in *H-RAS^G12V^* and *SCRIB^RNAi^/H-RAS^G12V^* cells compared with its levels in control cells and were reduced dramatically upon trametinib or combination drug treatment (Fig. 6A; additional replicates shown in Fig. S13A). pERK levels were also reduced upon trametinib and combination drug treatment in *SCRIB^RNAi^* and control cells (Fig. 6A; Fig. S13A). Surprisingly, however, we did not observe any substantial effect on pERK levels upon individual CU-3 treatment in any cell lines, suggesting that CU-3 does not affect Ras signalling. For the mTOR pathway, we found that trametinib alone and with CU-3 reduced pS6 levels in all cell lines; however, again unexpectedly, CU-3 did not substantially affect pS6 levels (Fig. 6B; Fig. S13B), suggesting that CU-3 also does not inhibit mTOR signalling in these cells. Trametinib has been reported to also show inhibition of mTOR signalling in some cell lines, although how this occurs is unclear (Vujic et al., 2015). Thus, CU-3 and trametinib treatment leads to an inhibition of Ras-MEK-ERK and mTOR-S6 signalling in all cell lines, which appears to be largely due to the effect of trametinib alone.

We then analysed the effect of the drug combination on other important signalling pathways involved in Ras-driven polarity-impaired tumour growth in *Drosophila* and mammalian cells: PI3K signalling-induced cell proliferation, JAK-STAT signalling-induced cell proliferation, Hippo signalling-induced negative tissue growth, and P38 stress-response pathways (Bunker et al., 2015; Doggett et al., 2011; La Marca and Richardson, 2020; La Marca et al., 2021; Norman et al., 2012; Richardson and Portela, 2017; Stephens et al., 2018). PI3K is a lipid kinase that can be upregulated by Ras signalling and positively regulates mTOR signalling (Carnero, 2010; Memmott and Dennis, 2009). To detect PI3K pathway signalling, we analysed the levels of phosphorylated AKT (pAKT), a PI3K target, relative to total AKT protein (Fig. S13C). pAKT levels were not greatly affected in *SCRIB^RNAi^/H-RAS^G12V^* cells relative to its levels in control cells and the combination drug treatment did not substantially affect pAKT levels. Thus, alterations in the PI3K signalling pathway are unlikely to be involved in the effect of trametinib and CU-3 on cell viability.

Analysis of the JNK family stress-response P38 pathway (Obata et al., 2000; Wagner and Nebreda, 2009) revealed that upon combination drug treatment, there was an ∼5-fold elevation in the ratio of the levels of the active phosphorylated isoform of P38 (pP38) to total P38 levels in *H-RAS^G12V^* cells and an ∼2-fold elevation in the ratio of pP38 levels to total P38 levels in *SCRIB^RNAi^/H-RAS^G12V^* cells relative to that in the corresponding DMSO-treated cells (Fig. 6C). The elevation of P38 signalling in only the *H-RAS^G12V^*-expressing cells, and not in the control or *SCRIB*-knockdown cells upon combination drug treatment, suggests that cellular changes induced by activated RAS responded to the trametinib and CU-3 combination drug treatment to lead to P38 activation. This result also suggests that synergy between trametinib and CU-3 may depend upon P38 activation in *SCRIB^RNAi^/H-RAS^G12V^* cells. As the P38 pathway is a regulator of cell quiescence (Adam et al., 2009; Chen et al., 2018; Soeda et al., 2017; Sosa et al., 2011; Whitaker and Cook, 2021; Yu-Lee et al., 2019) and apoptosis (Obata et al., 2000), the elevated pP38 levels may contribute to the decreased viability observed upon combination drug treatment of the *SCRIB^RNAi^/H-RAS^G12V^* cells by inducing cell quiescence and perhaps also cell death.

The JAK-STAT pathway is an inducer of cell growth and proliferation (Zoranovic et al., 2013); in *Drosophila*, its ligands are upregulated in *scrib* mutant cells (Bunker et al., 2015), it is required for *scrib* mutant cell proliferation (La Marca et al., 2021), it is upregulated in *scrib* mutant *Ras^V12^* tumours (Atkins et al., 2016) and it cooperates with Ras in tumour growth (Herranz et al., 2012; Wu et al., 2010). Analysis of phosphorylated STAT (pSTAT; active) levels relative to total STAT levels, as a readout of JAK-STAT pathway activity, in *SCRIB^RNAi^/H-RAS^G12V^* cells with or without combination drug treatment did not reveal any substantial changes compared to its levels in the control cells (Fig. S13D). Thus, the JAK-STAT signalling pathway is not likely to be involved in the viability of *SCRIB^RNAi^/H-RAS^G12V^* cells upon combination drug treatment.

The Hippo pathway, which negatively regulates tissue growth (Misra and Irvine, 2018; Richardson and Portela, 2017), is impaired in *Drosophila scrib* mutant tissue and its impairment is required for the proliferation and survival of *scrib* mutant cells (Chen et al., 2012; Doggett et al., 2011; Grzeschik et al., 2010); in mammalian cells, *SCRIB* loss results in impaired Hippo pathway signalling (Cordenonsi et al., 2011). Furthermore, in *Drosophila,* elevated EGFR-Ras-MAPK signalling or expression of human activated RAS mutations leads to impaired Hippo pathway signalling (Das et al., 2021; Reddy and Irvine, 2013). Thus, to determine whether the combination drug treatment affected the Hippo pathway in *SCRIB^RNAi^/H-RAS^G12V^* cells, we examined phosphorylated YAP (pYAP; inactive) levels relative to total YAP levels, as well as the levels of the YAP target, MYC (Archibald et al., 2015), relative to HSP70 protein levels. No substantial changes were observed in pYAP or MYC in *SCRIB^RNAi^/H-RAS^G12V^* cells relative to those in the control, nor upon combination drug treatment in *SCRIB^RNAi^/H-RAS^G12V^* cells or control cells (Fig. S13D,E). Thus, alterations in Hippo pathway signalling are unlikely to be involved in the viability of *SCRIB^RNAi^/H-RAS^G12V^* cells in response to combination drug treatment. Taken together, these analyses indicate that elevation of P38 signalling may be the most relevant in regard to the reduced viability of *SCRIB^RNAi^/H-RAS^G12V^* cells upon trametinib and CU-3 combination drug treatment.

## DISCUSSION

Combination drug therapy is becoming increasingly favoured as an anti-cancer therapy, allowing for lower doses of each drug to be used, decreasing unwanted side effects, and reducing the chance that drug resistance will occur (Al-Jundi et al., 2020; De Leo et al., 2020; Ferrari et al., 2020; Roskoski, 2017). However, identifying novel drug combinations that are effective against specific cancers can be a difficult prospect. In this regard, the *Drosophila* model has become a highly effective *in vivo* tool in discovering bioavailable efficacious drug combinations that are translatable to human cells and, in some cases, also clinically effective as anti-cancer therapies (Bangi et al., 2019, 2016, 2021; Levine and Cagan, 2016; Stickel et al., 2015). Using a *Drosophila* model of Ras-driven, polarity-impaired cancer, we undertook a specialised screen of chemical libraries and identified two compounds that showed synergy with sub-therapeutic doses of the MEK inhibitor trametinib, identifying the Polo-like kinase inhibitor volasertib, and the 5-HT2A/2C serotonin receptor and DGKα inhibitor ritanserin. Validating our approach, Polo-like kinase inhibitors have been extensively used in cancer therapy (Gjertsen and Schöffski, 2014; Shakeel et al., 2021; Zhang et al., 2021) and have also been explored as a potential therapy in combination with MEK inhibitors for the treatment of *N-RAS*-driven melanoma (Posch et al., 2015). Thus, we focused on characterizing the synergistic interaction between trametinib and ritanserin, revealing by pharmacogenetic analyses in *Drosophila* that the key target of ritanserin is Dgk (ortholog of mammalian DGKα/β/γ), rather than other *Drosophila* Dgk paralogs or serotonin receptors. Furthermore, we show that our findings in *Drosophila* are translatable to MCF10A human mammary epithelial cells harbouring oncogenic *H-RAS* and knockdown of the cell polarity gene *SCRIB*. We demonstrated that both ritanserin and another serotonin receptor and DGKα inhibitor, R-59-022, synergised with low doses of trametinib in *SCRIB^RNAi^/H-RAS^G12V^* cells (as well as in *SCRIB^RNAi^* cells and *H-RAS^G12V^* cells) – doses at which normal cells were not greatly affected, suggesting that these drug combinations could be used to specifically target Ras-driven and/or polarity-impaired cancer cells. Then, using a DGKα specific inhibitor, CU-3, we also demonstrated synergy with trametinib in MCF10A cells, but in this case, drug dose combinations affected the *SCRIB^RNAi^/H-RAS^G12V^* cells similarly to normal cells, suggesting that CU-3 would not be clinically useful in specifically targeting Ras-driven cancer cells. However, another compound that targets DGKα/β/γ isoforms (Compound A; unfortunately, to our knowledge, not yet commercially available) has been identified as highly effective in inducing apoptosis of a variety of cancer cells *in vitro* (Yamaki et al., 2019), and it would be interesting to determine whether this compound, as well as other newly identified DGK-specific inhibitors (Velnati et al., 2020, 2019), may have greater efficacy with trametinib in specifically targeting Ras-driven cancer cells relative to normal cells. Of the DGKα (and 5-HT2A/2C serotonin receptor) inhibitors we analysed, only ritanserin has been tested in human clinicals trials, but in the context of schizophrenia, cocaine and alcohol dependence, and migraines (Cornish et al., 2001; Johnson et al., 1996; Nappi et al., 1990; Wiesel et al., 1994), and it has not been marketed for clinical use. More recently, *in vitro* studies have shown that ritanserin induces cell death in the mesenchymal cell subtype of glioblastomas (Audia and Bhat, 2018; Olmez et al., 2018). Furthermore, it was shown that ritanserin can synergise with irradiation and with imatinib, a tyrosine kinase inhibitor, to decrease cell proliferation of mesenchymal glioblastoma cells (Olmez et al., 2018). Similarly, the DGKα-specific inhibitor Amb639752 promotes restimulated apoptosis of cells *in vitro* and in animal models of X-linked lymphoproliferative disease type 1, which have elevated DGKα activity (Velnati et al., 2021a,b, 2020). These studies, along with our own findings, suggest that ritanserin as well as other DGKα inhibitors would be good candidates to be developed clinically as anti-cancer drugs and to be used in combination with trametinib or other EGFR-Ras pathway inhibitors, particularly in Ras-driven cancers.

**Fig. 7.**
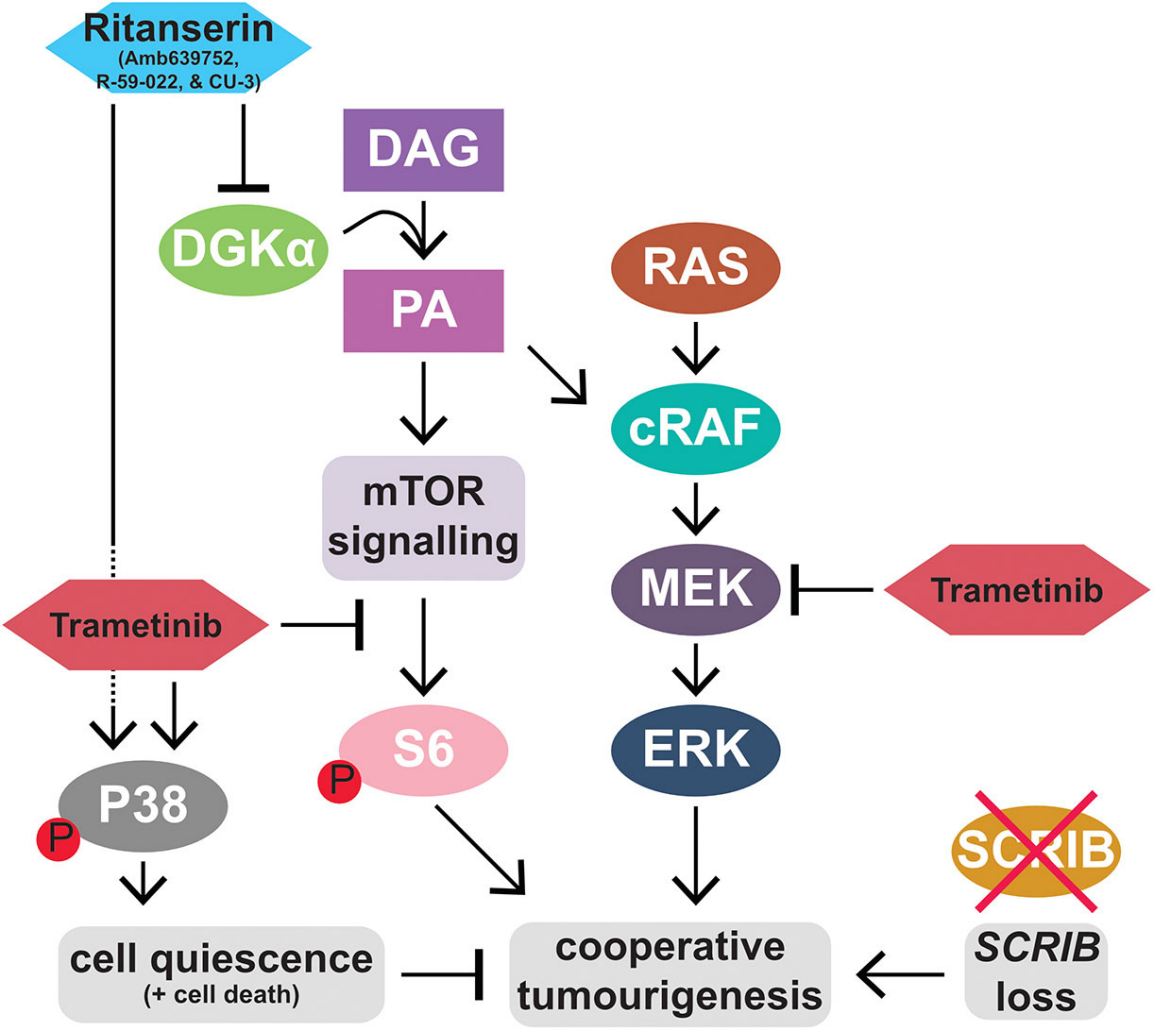
**Model of DGKα involvement in tumourigenesis driven by *SCRIB* loss and RAS activation, and the effect of trametinib and DGKα inhibitors.** Activated RAS induces signalling via the cRAF-MEK-ERK cascade, promoting hyperplastic tissue growth. The additional inhibition/loss of *SCRIB* results in cooperative tumourigenesis. Trametinib, a MEK inhibitor, can modulate this signalling and leads to a reduction in tumour growth. Our data show that trametinib synergises with ritanserin, Amb639752, R-59-022 and CU-3 to strongly inhibit tumour growth. These drugs act by inhibiting the activity of DGKα, which catalyses the conversion of diacylgycerol (DAG) into phosphatidic acid (PA). Evidence suggests that PA stimulates cRAF-MEK-ERK signalling, as well as mTOR signalling. However, we did not find that CU-3 independently inhibited these pathways, whereas trametinib inhibited both. Instead, we found that in *H-RAS^G12V^*-expressing human epithelial cells, CU-3 synergised with trametinib to activate the P38 stress-response pathway. Activated P38 may reduce cell viability and inhibit tumour growth by inducing cell quiescence and perhaps by increasing cell death. RAS, RAS proto-oncogene, GTPase; cRAF, Raf-1 proto-oncogene, serine/threonine kinase; MEK, mitogen-activated protein kinase kinase 7; ERK, mitogen-activated protein kinase 1; SCRIB, scribble cell polarity protein; mTOR, mechanistic target of rapamycin kinase; DGKα, diacylglycerol kinase α; S6, ribosomal protein S6 kinase B1; P38, mitogen-activated protein kinase 14.

DGKα is considered an oncogene, being upregulated in many cancers, and promotes cell proliferation and cell survival (Chen et al., 2019; Fazio et al., 2020; Mérida et al., 2017; Sakane et al., 2021). Elevated DGKα activity phosphorylates DAG to form PA, promoting signalling pathways regulated by PA and attenuating those regulated by DAG (Andresen et al., 2002; Foster et al., 2014; Zhang and Du, 2009) (Fig. 7). PA upregulates Ras-RAF-MEK-ERK signalling by increasing the binding of cRAF to the endosomal membranes, where it interacts with active RAS (RAS-GTP) and forms a scaffold with MEK and ERK, thereby enabling Ras pathway activation and promoting cell proliferation and survival (Andresen et al., 2002). PA also promotes mTOR activity by enabling the interaction of mTOR with the Raptor adaptor protein to form the mTORC1 complex, and with the Rictor adaptor protein to form the mTORC2 complex (Fang et al., 2001; Toschi et al., 2009) (Fig. 7). DGKα has also been reported to positively regulate mTOR transcription through a mechanism involving phosphodiesterase 4A1 and cyclic-AMP (Dominguez et al., 2013). mTOR, through its promotion of protein synthesis, plays an important role in stimulating cell growth and proliferation (Saxton and Sabatini, 2017). Thus, it was expected that by inhibiting DGKα activity and decreasing PA levels, Ras and mTOR signalling will be inhibited, thereby inhibiting cell growth, proliferation and survival. Surprisingly, we found that CU-3 treatment did not inhibit Ras or mTOR signalling in any of the cell lines, although treatment with trametinib inhibited both pathways. The mTOR pathway has previously been shown to be important for polarity-impaired Ras-driven tumour growth in *Drosophila* (Willecke et al., 2011), and its inhibition by trametinib that we observed in our study is likely to contribute to the G1 cell cycle arrest observed upon trametinib treatment.

Our signalling pathway analyses reveal that none of the pathways we analysed (Ras, mTOR, PI3K, JAK-STAT, Hippo or P38) were affected by sole inhibition of DGKα by CU-3. Whether other pathways known to be regulated by DGKα activity (Sakane et al., 2021) are important targets in *SCRIB^RNAi^/H-RAS^G12V^* cells will be important to investigate. One such pathway, activated by PA (which is elevated by DGKα activity), is atypical protein kinase C ζ (PKCζ)-nuclear factor κ B (NFκB) signalling, which promotes cell survival (Kai et al., 2009; Sakane et al., 2021). Atypical protein kinase C (aPKC) is another cell polarity regulator important in establishing and maintaining the apical-basal polarity of epithelial cells (Tepass, 2012; Vorhagen and Niessen, 2014) and negatively regulating the Hippo signalling pathway that negatively regulates tissue growth in both mammalian cells (Archibald et al., 2015) and *Drosophila* (Doggett et al., 2011; Grzeschik et al., 2010). As *Drosophila* Ras-driven, polarity-impaired tumours have unrestrained activity of aPKC [an important factor in inducing tumour growth (Leong et al., 2009)], which occurs via Hippo pathway inhibition (Doggett et al., 2011), it is possible that by inhibiting DGKα, the elevated aPKC activity and Hippo pathway impairment would be rescued. However, our analyses of *SCRIB^RNAi^/H-RAS^G12V^* human epithelial cells treated with CU-3 and trametinib did not reveal any substantial affect on Hippo signalling, but whether it affects PA-PKCζ-NFκB signalling would be interesting to determine.

Another important aspect of DGKα activation is the depletion of DAG levels, which has the potential to impact various signalling pathways (Griner and Kazanietz, 2007; Mérida et al., 2008). DAG binds to and activates the C1 domain of protein kinase C family proteins, as well as other non-kinase proteins, including GTPase regulators (Colón-González and Kazanietz, 2006; Griner and Kazanietz, 2007; Oliva et al., 2005; Wang and Kazanietz, 2006). Protein kinase C family members function downstream of growth factor receptors to promote signalling (Oliva et al., 2005), and therefore, DGK activation and decreased DAG would limit their activation. One of the non-kinase proteins activated by DAG is RAC-GAP, which is involved in the inactivation of the RAC-GTPase (Wang and Kazanietz, 2006), an important regulator of the actin cytoskeleton and signalling from cell-cell adhesion junctions (Bosco et al., 2009; Ratheesh et al., 2013). DGK activation and concomitantly lower DAG would therefore reduce RAC-GAP activity and increase RAC activity, which might contribute to *SCRIB^RNAi^/H-RAS^G12V^* tumourigenic properties, as *Drosophila* studies have shown that Rac activates JNK signalling (Brumby et al., 2011; Ma et al., 2015), a key factor in promoting *scrib* mutant Ras-driven tumourigenesis by inhibiting differentiation and promoting invasion (Igaki et al., 2006; Leong et al., 2009; Uhlirova and Bohmann, 2006). Dgk inhibition, by increasing DAG and activating Rac-GAP, would be expected to reduce Rac activity and JNK activity and inhibit *scrib* mutant Ras-driven tumourigenic properties. The investigation of the involvement of DAG-regulated signalling pathways in *SCRIB^RNAi^/H-RAS^G12V^* tumour growth and the effect of inhibition of DGKα on these pathways will be important in providing a full picture of the involvement of DGKα in Ras-driven cancers.

Importantly, our study revealed that the activity of the P38 stress-response pathway was increased in *H-RAS^G12V^* and *SCRIB^RNAi^/H-RAS^G12V^* cells upon treatment with trametinib and CU-3. As the P38 pathway is involved in the apoptosis of the *SCRIB*-impaired cells in a cell competition setting (Norman et al., 2012) and is known to regulate apoptosis in human cells (Obata et al., 2000), it is possible that elevated P38 activity promotes apoptosis of the drug-treated human cells. However, we did not observe any substantial cell death at either 24 or 72 h post drug treatment, although it is possible that it might have occurred later. The inhibition of cell viability (measured by ATP levels) observed in the CellTiter-Glo BLISS assays upon combination drug treatment is more likely to be explained by the activation of P38 inducing cell quiescence (G0 cell cycle state), as has been previously documented (Adam et al., 2009; Chen et al., 2018; Soeda et al., 2017; Sosa et al., 2011; Whitaker and Cook, 2021; Yu-Lee et al., 2019). Mechanistically, activated P38 signalling has been described as inducing quiescence by upregulating the P53 tumour suppressor protein and decreasing the expression of the mitogenic transcription factors, c-Jun and FoxM1 (Sosa et al., 2011). Cell quiescence is due to elevated expression of cell cycle inhibitors, such as the G1 cyclin-dependent kinase inhibitors, P21, P27 and P57, and results in transcriptional and metabolic changes (Marescal and Cheeseman, 2020). Cellular ATP levels are substantially reduced in quiescent cells (Marescal and Cheeseman, 2020), which would be expected to lead to a lower cell viability level in the CellTiter-Glo assay. Although the activation of P38 and its expected induction of quiescence in the drug-treated *SCRIB*-knockdown *H-RAS^G12V^*-expressing cells and *H-RAS^G12V^*-expressing cells could explain the synergy between trametinib and DGKα inhibitors, precisely how the combination drug treatment results in elevated P38 activity in these cells remains to be determined.

In summary, our analyses have revealed that Dgk/DGKα plays an important role in Ras-driven polarity-impaired tumour growth in both the *Drosophila* model and human epithelial cell lines. Our findings raise many questions that remain to be investigated, such as whether DGKα activity is elevated in Ras-driven polarity-impaired cells and, if so, how this occurs, and whether increased PA signalling or decreased DAG signalling provide the critical function of DGKα in tumour growth. However, as DGKα is upregulated and oncogenic in various human cancers (Chen et al., 2019; Fazio et al., 2020; Mérida et al., 2017; Sakane et al., 2021), the findings from our study suggest that Ras-driven polarity-impaired cancers may be particularly dependent on DGKα for tumour survival and, therefore, that DGKα inhibitors and Ras pathway inhibitors would be a highly effective drug combination for anti-cancer therapy in these cancers. DGKα inhibitors have previously been considered for development as anti-cancer therapy, not only for their effect on the cancer but also on the T-cell anti-cancer immune response (Arranz-Nicolás and Mérida, 2020; Sakane et al., 2008, 2016). Our findings suggest that combining Ras pathway inhibitors with DGKα inhibitors may provide even greater efficacy against Ras-driven cancers, as well as decrease unwanted side effects and reduce the development of drug resistance.

## MATERIALS AND METHODS

### *Drosophila melanogaster* stocks and husbandry

Fly stocks used in this study are detailed in Table S1. Unless otherwise indicated, animals were maintained and crosses were undertaken on a standard cornmeal/molasses/yeast medium within temperature-controlled incubators at 25°C.

### Generation of larvae for screening

Approximately 450 *eyFLP, UAS-GFP;; tub-GAL4, FRT82B, tub-GAL80/TM6B, Tb-RFP* females were crossed to approximately 150 *UAS-Ras85D^V12^, FRT82B, scrib^1^/TM6B* males. Crosses were undertaken in cages containing apple juice agar plates [‘lay plates’ – 35 g agar (Amresco, J637) and 20 g sucrose (Sigma-Aldrich, S-0389) were dissolved in 1 l H_2_O by microwaving, 250 ml apple juice (Spring Valley) was added, followed by incubation at 60°C for 1-2 h, then 25 ml Tegosept solution (100 g methylhydroxybenzoate in 1 l absolute ethanol) was added and the solution was set in 10 cm Petri dishes]. On the lay plates, larvae/flies were fed with yeast paste [∼100 g compressed yeast (Lesaffre) in H_2_O to thickened consistency]. A laying period of 7-14 h was used. Early third-instar larvae (as indicated by size) with the genotype *eyFLP, UAS-GFP; UAS-Ras85D^V12^/+; FRT82B, scrib^1^/tub-GAL4, FRT82B, tub-GAL80* were collected at 48-60 h after egg laying by rinsing the lay plate with tap water before tipping the contents through a small sieve. Larvae were then selected on plates for compound screening based on the presence/absence of physical markers (observed GFP expression in the head region and lack of TM6B-RFP markers) using a SteREO Discovery.V8 microscope (Zeiss). This method was adapted from Willoughby et al. (2013).

### Compound screening

Approximately 2 mg of instant *Drosophila* medium (Southern Biological, CM4) was added to each well of a deep-well 96-well plate (Nunc, 260252). This medium was reconstituted with 240 µl yeast solution [14 g dried yeast (Tandaco) dissolved in 250 ml H_2_O by microwaving for 10 min to inactivate yeast, and stored at 4°C], which contained the compound(s) of interest (File S1), or a DMSO control at 0.5% v/v. Larvae (generated as above) were then added to each well of the 96-well plate at a density of ∼7 per well. The plate was sealed with wire mesh and Perspex (containing 96 holes for airflow), and incubated at 25°C for 5 days (∼120 h). An unsealed container of water was incubated next to the plate to maintain humidity and prevent desiccation. Sucrose solution (30% w/v sucrose in H_2_O) was then added to each well and the larvae dislodged from the food via agitation. Additional sucrose solution was added to each well until a convex meniscus formed, allowing larvae to float to the top of the well and into the focal plane. The plate was then imaged two wells at a time using the SteREO Discovery.V8 microscope with Zen 2012 software (Zeiss). The resulting images were stitched together using Photoshop (various editions, Adobe). The stitched image was then thresholded using Fiji (Schindelin et al., 2012), with the pixel intensity threshold determined as the value that first eliminated any background signal. This resulted in a binarised image where white pixels represented GFP-positive tumourigenic tissue. A white pixel count was then performed for each well using the Fiji ‘Analyse Particles’ function (Schindelin et al., 2012). To maximise consistency results, an ImageJ Macro was written, which automatically performed white pixel counts automatically on areas corresponding to the plate wells using a nested FOR loop (File S4). Areas of white pixels smaller than 2[Supplementary-material sup1]pixels were excluded from the analyses as noise. The data was then exported to Excel (Microsoft), and the pixel count for each well was divided by the number of larvae in the respective well, producing the mean GFP-positive pixel area per organism for each well as a representation of tumour size.

### Compounds

Compounds utilised in this study were selected on the basis of their identification as potential hits in a primary compound screen (File S1). The primary compound screen was performed as described above. Each compound derives from one of four specialised screening libraries (‘epigenetic library’, ‘kinase library’, ‘targeted agents library’ and ‘FDA-approved known drug library’) obtained from Hélène Jousset Sabroux and Kym Lowes [Walter and Eliza Hall Institute (WEHI), Australia]. All compounds were dissolved in DMSO, then in 2 ml yeast solution, to obtain the desired concentrations (see File S1, Table 1 and relevant figures), and such that the final concentration of DMSO was not greater than 0.5% v/v [which we have shown previously to not effect the viability of the larvae (Willoughby et al., 2013)]. The concentrations of compounds listed are the final concentrations of the compounds in the food; however, we do not know the concentrations of compounds that the larval cells were exposed to. Additional compounds were obtained from the following sources: R-59-022 (Cayman Chemical Company, 16772), Amb635792 (Ambinter) and CU-3 (MedChemExpress, HY-121638A).

### Statistical analyses

For each compound treatment, for each well, the mean GFP-positive pixel area per organism was divided by the mean GFP-positive pixel area per organism of all DMSO-treated wells, obtaining the mean GFP pixel intensity per organism per well normalised to DMSO. These values were analysed statistically using Prism (GraphPad), with the particular tests employed for each analysis detailed in the respective figure legends.

### GFP-positive area to larvae pixel area ratio analysis

Binarised plate images and their respective unaltered plate images were imported into Photoshop 2020 (Adobe). For two larvae per well, larval size (area in pixels) was determined using the ‘Lasso’ tool. The GFP-positive tumour size (area in pixels) for the respective tumour(s) of each larvae measured were determined using the ‘Magic Wand’ tool. The GFP-positive pixel area to larva pixel area ratio was then calculated for each animal by dividing the size of the tumour by the size of the respective larva.

### RNA extraction, cDNA synthesis and qRT-PCR

RNAi lines were crossed to *hsFLP; Actin*≫*GAL4, UAS-GFP* and raised at 25°C. Whole adults or pupae (as some crosses were lethal at the pupal stage) were homogenised (*n*=∼10 animals per genotype) in 1× PBS. RNA extraction and cDNA synthesis were performed as previously described (La Marca et al., 2019). Real-time quantitative reverse transcription PCR (qRT-PCR) was performed using a Power SYBR Green PCR Master Mix (Applied Biosystems, 4367659) and a QuantStudio 12K Flex Real-Time PCR System (Applied Biosystems). The data were normalised to expression of the housekeeping genes *Gapdh2* and *RpL32*. The primer sequences used are listed in Table S2 and were obtained from Integrated DNA Technologies.

### Tissue imaging

RNAi lines were crossed to both *eyFLP;; Act*≫*GAL4, UAS-GFP/TM6B* (EAG) and *eyFLP; UAS-Ras85D^V12^, UAS-dlg1 RNAi/CyO tub-GAL80; Act*≫*GAL4, UAS-GFP/TM6B* (EAGRD), and incubated at 25°C for ∼7 days. *UAS-luciferase RNAi* was used as a non-targeting control. Third instar larvae were dissected in 1× PBS (Amresco, E703), fixed with 4% paraformaldehyde (Alfa Aesar, 43368) in 1× PBS with 0.1% Triton X-100. Samples were incubated in DAPI (stock prepared at 1 μg/ml, used at 1:1000; Sigma-Aldrich, D9542) and phalloidin-tetramethylrhodamine isothiocyanate solution (used at 0.3 µM, Sigma-Aldrich, P1951) to mark DNA and F-actin, respectively. Samples were imaged via confocal microscopy using an LSM 780 microscope (Zeiss), and the images processed using Zen 2012 (Zeiss) and Photoshop (Adobe). Imaris (Bitplane) was used to measure the volumes of the eye-antennal discs and brain lobes, which were identified using DAPI-positive tissue.

### MCF10A cell culture

Stably transformed MCF10A cells were used for cell culture experiments with the following genotypes: *MSCV-Scramble* (control), *MSCV-shSCRIB7*, *H-RAS^G12V^-Scramble* and *H-RAS^G12V^-shSCRIB7* (Dow et al., 2008)*.* MCF10A cells were maintained at 37°C and 5% CO_2_ in Dulbecco's modified Eagle medium/F12 (Thermo Fisher Scientific, 10565018) with donor horse serum (20 ng/ml; Thermo Fisher Scientific, 26050088), EGF (100 ng/ml; Preprotech, AF-100-15) and cholera toxin (100 ng/ml; List Labs, 100B).

### Western blotting

For the western blotting experiments, MCF10A cells were plated at 140,000 cells/well of a six-well plate (Costar, 3506) and incubated at 37°C with 5% CO_2_. The next day, the medium was removed and DMSO or trametinib (1.1515 µM) and CU-3 (10 µM) were applied in 1.5 ml medium. Cells were treated in drugs for 24 or 48 h, and for collection, the cells were washed with tissue culture-grade PBS (TC-PBS), dissociated with 1× trypsin (Lonza, BE02-007E) and washed again with TC-PBS, and the pellet was collected and snap frozen, before storage at −20°C.

Protein was isolated by incubation for 30 min on ice in RIPA buffer (150 mM NaCl, 0.1% w/v SDS, 1% Triton X-100, 0.5% sodium deoxycholate, 50 mM Tris-HCl buffer pH 8.0) with cOmplete Protease Inhibitor Cocktail (Roche, 11836153001) and PhosSTOP (Roche, 04906845001) as a phosphatase inhibitor. Cell lysates were then centrifuged at 16.2 ***g*** for 10 min at 4°C, and the supernatant stored at −20°C as needed. For all samples, to determine protein concentrations, a Pierce BCA Protein Assay Kit (Thermo Fisher Scientific, 23225) was used. Then, 4× Laemmli buffer (0.25 M Tris-HCl buffer, 40% glycerol, 8% SDS and 0.1% w/v bromophenol blue) with 1:10 β-mercaptoethanol (BDH, 441433A) was added to protein lysates and samples were boiled for 5 min. Equal quantities of protein (10-20 µg) were loaded on a 4-12% NuPAGE Bis-Tris gel (Thermo Fisher Scientific, NP0335BOX) and run at 120-150 V. Precision Plus Protein Kaleidoscope Prestained Protein Standard (Bio-Rad, 1610375) was used as a molecular mass marker. Gels were transferred to an iBlot Transfer Stack nitrocellulose membrane (Life Technologies, IB23001) using an iBlot2 Transfer device as per the manufacturer's protocols. Membranes were blocked in 5% bovine serum albumin (BSA) (Sigma-Aldrich, A7906) or skim milk powder in 1× TBS containing 0.1% Tween-20 (Sigma-Aldrich, P1379) (TBST) for 1 h at room temperature with gentle agitation. Membranes were then incubated in primary antibodies (Table S3) diluted in 5% BSA in 1× TBST overnight at 4°C with rolling agitation. Membranes were then washed four times for 10 min each in 1× TBST, incubated in the appropriate secondary antibody diluted in 5% BSA or skim milk powder in 1× TBST for 1 h at room temperature, then washed again four times for 10 min each in 1× TBST. The secondary antibodies used were goat anti-mouse Ig, human ads-HRP (Southern Biotech, 1010-05) and goat anti-rabbit Ig, human ads-HRP (Southern Biotech, 4010-05). Immobilon Forte Western HRP Substrate (Merck Millipore, WBLUF0500) was used to resolve the staining before the membranes were imaged on a ChemiDoc MP Imaging System (Bio-Rad). As needed, membranes were incubated in HRP inactivation solution (0.2% NaN_3_ in 1× PBS for 30 min with agitation) or mild stripping solution (1.5% w/v glycine, 0.1% w/v SDS, 1% v/v Tween-20, pH 2.2, in H_2_O) to re-probe the membrane. Western blot images were quantified using Fiji (Schindelin et al., 2012). To visualise these quantifications, the protein expression levels were each normalised to their respective levels in the DMSO-treated control cell line, then plotted as ratios relative to their corresponding control protein values using Prism.

### IC_50_ and BLISS assays

MCF10A cells were plated at 1000 cells/well of a 96-well flat-bottom white plate (Greiner Bio CELLSTAR, 655083) and incubated at 37°C with 5% CO_2_. Wells at the border of the plate were filled with H_2_O to prevent evaporation. The next day, cells were treated with ritanserin, trametinib, R-59-022, CU-3 or drug combinations at the concentrations indicated in the relevant figures. Cells were then incubated for a further 72 h. The medium was removed from wells and cells were washed with TC-PBS. Then, 40 µl TC-PBS was added to each well, followed by 40 µl CellTiter-Glo 2.0 Viability Assay Reagent (Promega, G9241), which measures metabolically active cells. Plates were incubated on an orbital shaker in darkness for 5 min at room temperature before luminescence was read on a CLARIOstar Plus plate reader (BMG Labtech). Luminescence readings were normalised to wells containing DMSO-treated cells. For IC_50_ assays, cells were plated in duplicate, two to three independent experiments were performed, and each IC_50_ value was calculated in Prism using nonlinear regression (curve fit) analysis. For BLISS assays, two to four independent experiments were performed and the average reading across experiments was used to build the final synergy scores. BLISS synergy scores were calculated for each well using standard methods (Bliss, 1939) and three-dimensional plots generated in Microsoft Excel.

### Cell cycle and cell death assays

For the cell cycle assays, MCF10A cells were plated at 8000 cells/well of a 96-well flat bottom plate (Falcon, 353072) for the 24 h treatment, and 4000 cells/well for the 72 h treatment. For the cell death assays, MCF10A cells were plated at 20,000 cells/well of a 12-well flat bottom plate (Costar, 3512) for the 24 h treatment, and 10,000 cells/well for the 72 h treatment. Cells were incubated at 37°C with 5% CO_2_. The next day, cells were treated with ritanserin, CU-3, trametinib, or the DGK inhibitors in combination with trametinib at the IC_50_ concentrations determined for the control cells. Cells were then incubated for 24 or 72 h before being processed. For the cell cycle assays, to fix and permeabilise the cells, an eBioscience Foxp3/Transcription Factor Staining Buffer Kit (Thermo Fisher Scientific, 00-5523-00) was used according to the manufacturer's instructions, with a modified permeabilisation step to be >24 h. For both the cell cycle and cell death assays, cells were finally resuspended in TC-PBS with DAPI (1:1000) to mark cell cycle stages or dead cells, respectively. Both the cell cycle and cell death assay samples were analysed using an LSR IIW FACS machine (BD Biosciences). Data were quantified using FlowJo (BD Biosciences) and analysed in Prism.

## Supplementary Material

10.1242/dmm.049769_sup1 Supplementary information
