# Supplementary material for: A Drosophila chemical screen reveals synergistic effect of MEK and DGKα inhibition in Ras-driven cancer
Source: Dis Model Mech. 2023 Apr 3;16(3):dmm049769. doi: 10.1242/dmm.049769 (PMC10110402; doi:10.1242/dmm.049769)
Supplement: Supplementary information [file dmm-16-049769-s1.pdf]

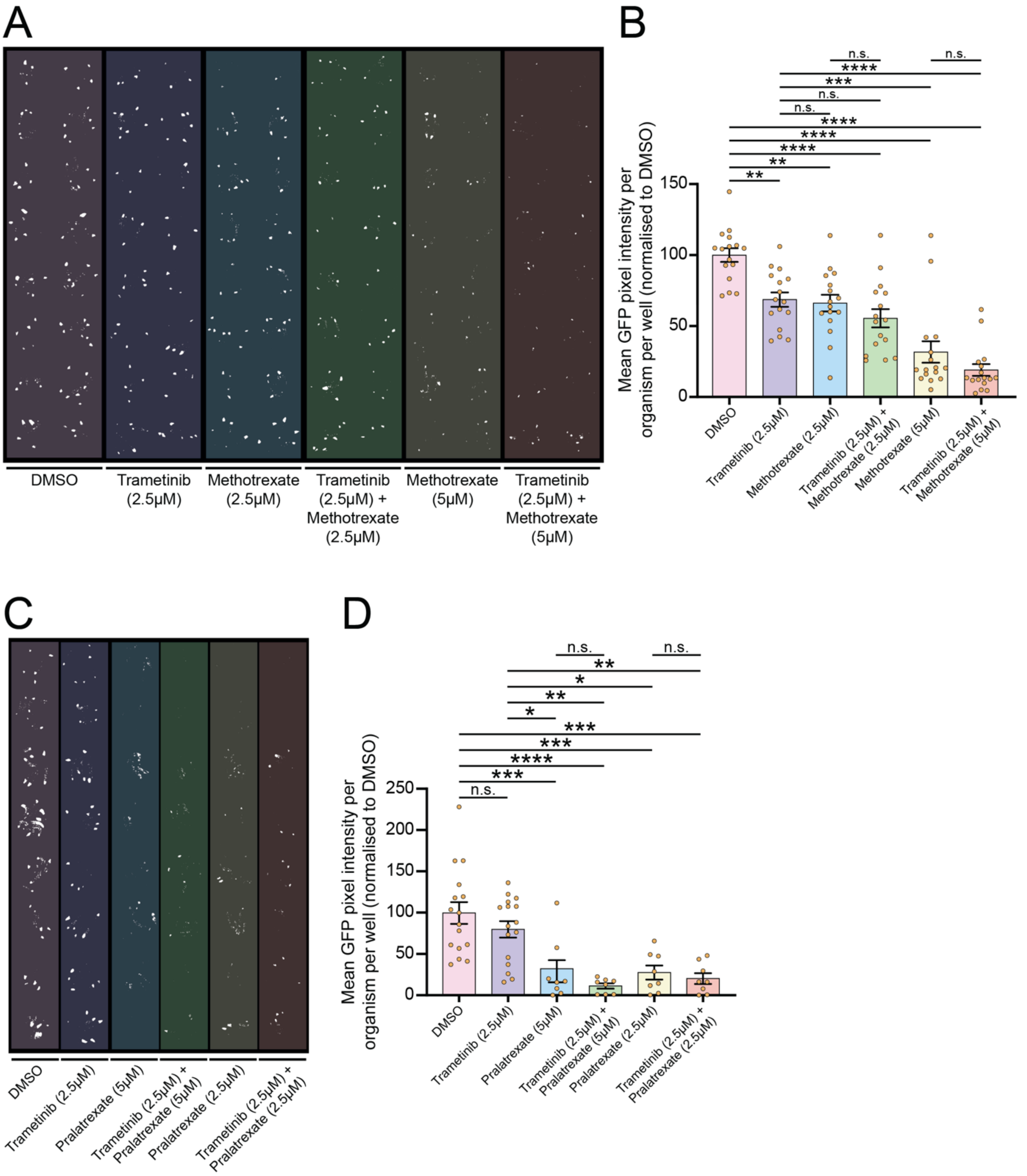

Fig. S1. Methotrexate and Pralatrexate reduce *scrib⁻/Ras^{V12}* tumour size independently of Trametinib.

(A) Binarized image of a drug test plate where larvae were treated with Trametinib in combination with the folate inhibitor, Methotrexate. (B) Quantifications of GFP pixel

Disease Models & Mechanisms • Supplementary information

intensity revealed that Methotrexate reduces tumour size, but does not synergise with Trametinib, regardless of the concentration tested. Data from 1 experiment with 16 replicate wells for each treatment. (C) Binarized image of a drug test plate where larvae were treated with Trametinib in combination with another folate inhibitor, Pralatrexate. (D) Quantifications of GFP pixel intensity revealed that Pralatrexate also reduces tumour size, but does not synergise with Trametinib, regardless of the concentration tested. Data from 1 experiment with 8 replicate wells for each treatment. Statistical tests used were one-way ANOVAs with Tukey's multiple comparisons. Error bars represent S.E.M. ** = $p<0.01$, *** = $p<0.001$, **** = $p<0.0001$.

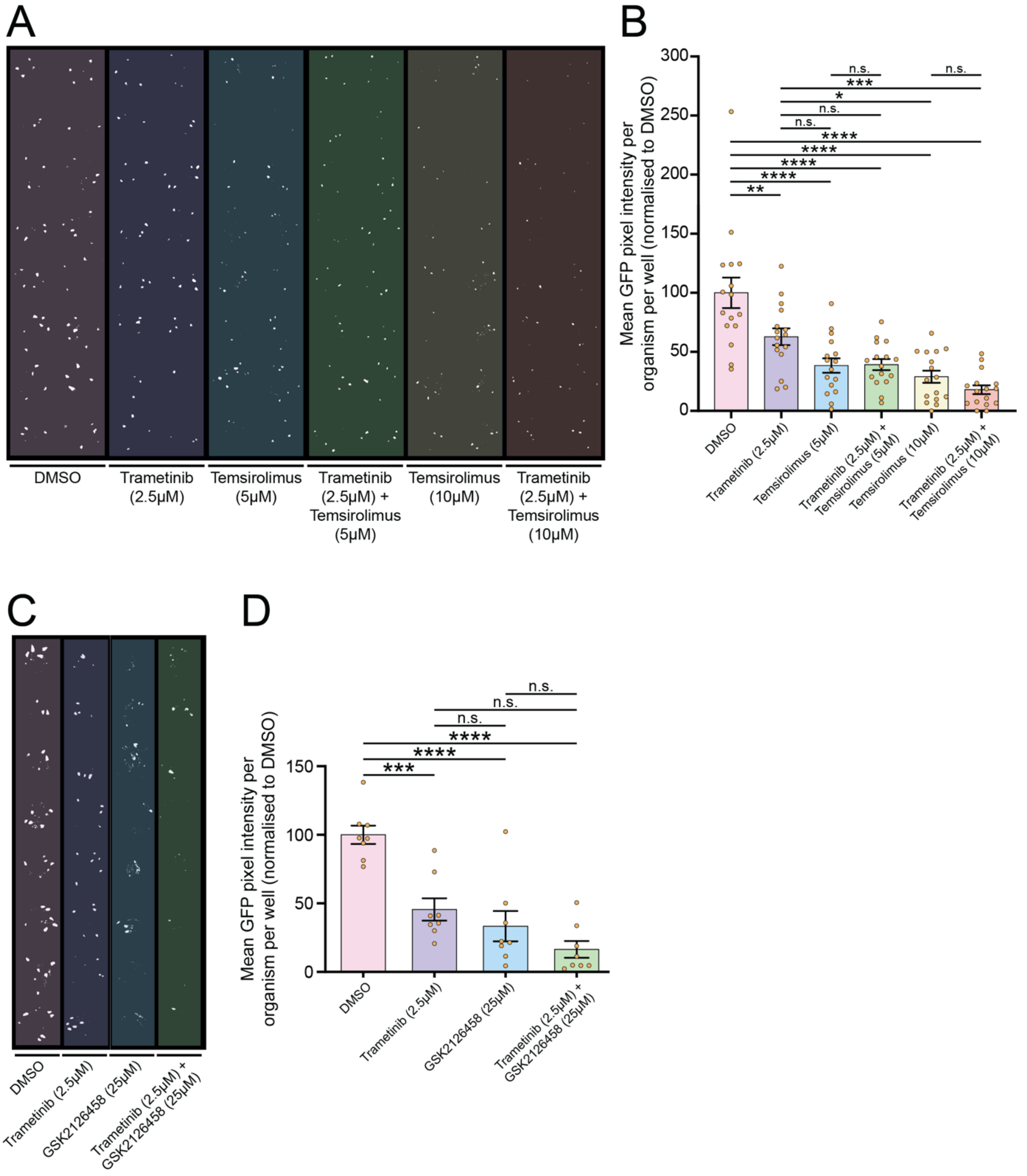

Fig. S2. Temsirolimus and GSK2126458 reduce *scrib⁻/Ras^{V12}* tumour size independently of Trametinib.

(A) Binarized image of a drug test plate where larvae were treated with Trametinib in combination with the mTOR inhibitor, Temsirolimus. (B) Quantifications of GFP pixel intensity revealed that Temsirolimus reduces tumour size, but does not synergise with

Disease Models & Mechanisms • Supplementary information

Trametinib, regardless of the concentration tested. Data from 1 experiment with 16 replicate wells for each treatment. (C) Binarized image of a drug test plate where larvae were treated with Trametinib in combination with the PI3K inhibitor, GSK2126458. (D) Quantifications of GFP pixel intensity revealed that GSK2126458 also reduces tumour size, but does not synergise with Trametinib. Data from 1 experiment with 8 replicate wells for each treatment. Statistical tests used were one-way ANOVAs with Tukey's multiple comparisons. Error bars represent S.E.M. ** = $p<0.01$, *** = $p<0.001$, **** = $p<0.0001$.

Disease Models & Mechanisms • Supplementary information

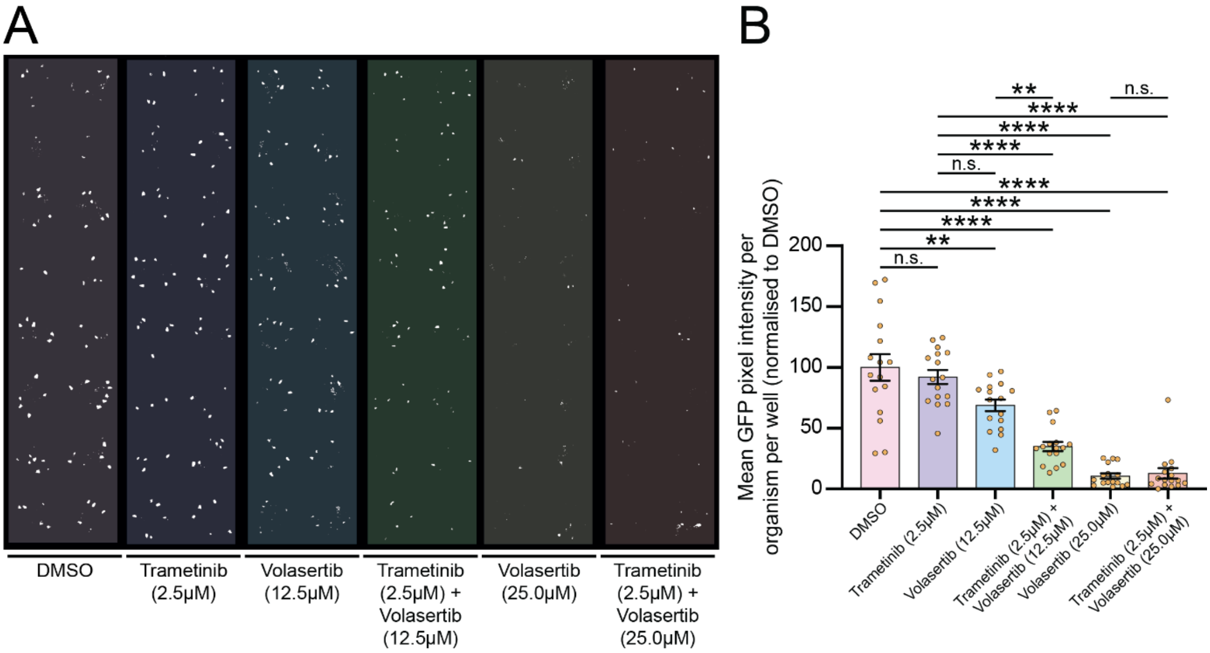

Fig. S3. Volasertib shows synergy with Trametinib in reducing the tumour size in *scrib⁻/Ras*^{V12} tumour-bearing larvae.

(A) Binarized image of a drug test plate where larvae were treated with Trametinib in combination with the Polo-like kinase inhibitor, Volasertib. (B) Quantifications of GFP pixel intensity revealed that Volasertib synergised with Trametinib at final concentrations of 12.5μM and 2.5μM, respectively, to significantly reduce tumour size compared to DMSO and the single-agent controls. However, increasing the Volasertib concentration to 25μM led to a reduction of the tumour size independent of Trametinib. Data from 1 experiment with 16 replicate wells for each treatment. Statistical test used was a one-way ANOVA with Tukey's multiple comparisons. Error bars represent S.E.M. ** = p<0.01, **** = p<0.0001.

Disease Models & Mechanisms • Supplementary information

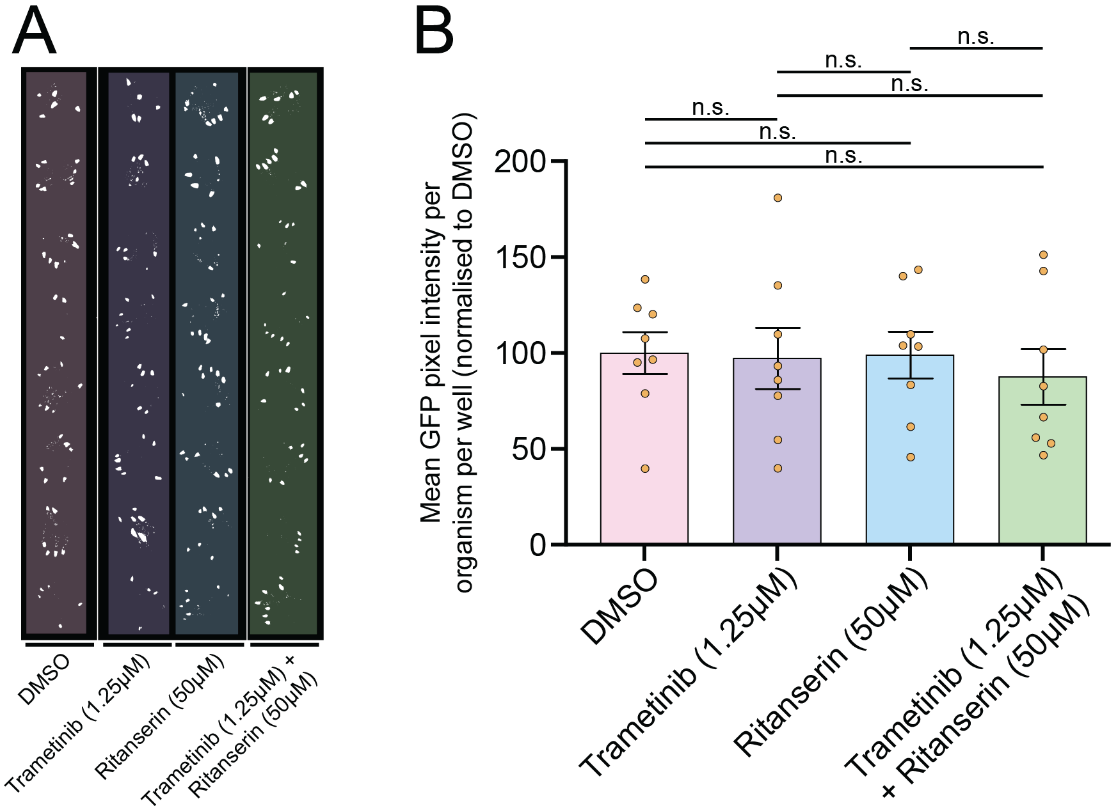

Fig. S4. Reduced Trametinib concentration precludes the synergistic effect.

(A) Binarized image of a drug test plate where larvae were treated with Trametinib in combination with the Ritanserin, using reduced concentrations of Trametinib. (B) Quantifications of GFP pixel intensity revealed that lower Trametinib concentrations remove the synergistic effects observed together with Ritanserin. Data from 1 experiment with 8 replicate wells for each treatment. Statistical tests used were one-way ANOVAs with Tukey's multiple comparisons. Error bars represent S.E.M. * = p<0.05, ** = p<0.01.

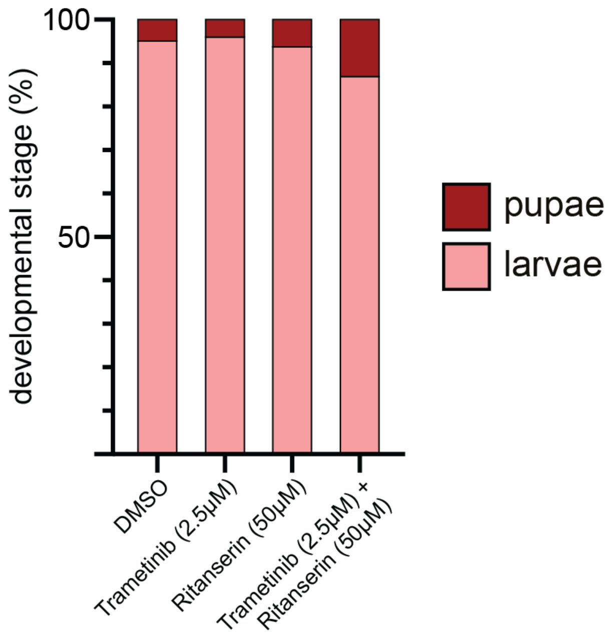

Fig. S5. Pupation quantification from initial drug plate experiments reveal the combination treatment promotes increased development.
Quantification of the developmental stage of the larvae at the endpoint of the various drug plate experiments conducted over the course of this study (~700 animals for each treatment). There was little difference in the proportion of animals that underwent pupation when treated with either DMSO (~6%), Trametinib (~5%), or Ritanserin (~7%) alone. However, the combination of Trametinib and Ritanserin resulted in a doubling of the percentage of animals undergoing pupation (~14%), suggesting the combination treatment is necessary to rescue larval development.

Disease Models & Mechanisms • Supplementary information

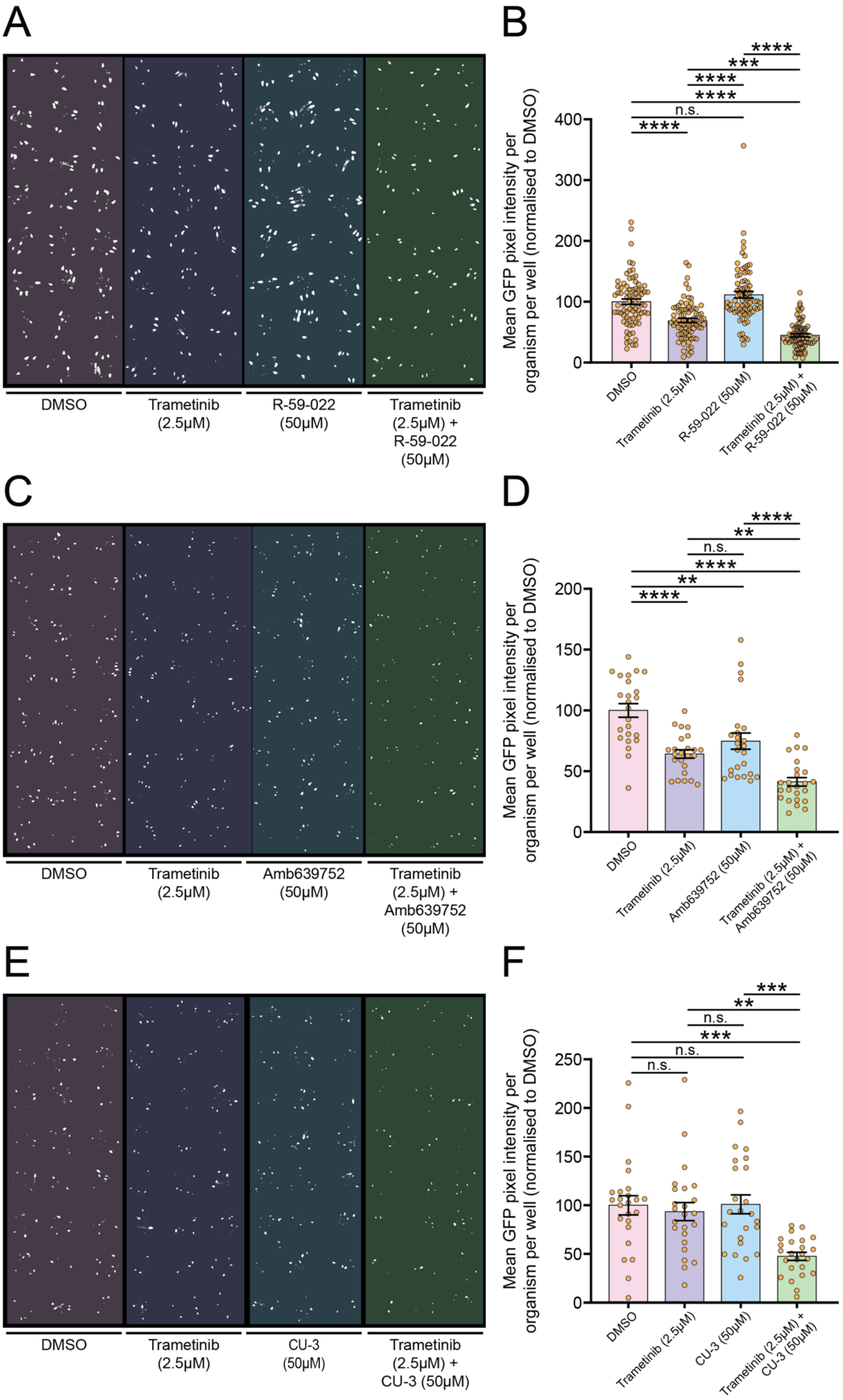

Disease Models & Mechanisms • Supplementary information

Fig. S6. Other DGK and 5-HT serotonin receptor inhibitory drugs replicate the *scrib⁻/Ras*^{V12} tumour reduction obtained using Ritanserin. (A) Binarized image of a drug test plate where larvae were treated with Trametinib in combination with the DGK and 5-HT inhibitor, R-59-022. (B) Quantifications of GFP pixel intensity revealed that R-59-022, like Ritanserin, results in a synergistic reduction in tumour size together with Trametinib. Data from 4 experiments, each with 16-24 replicate wells for each treatment. (C) Binarized image of a drug test plate where larvae were treated with Trametinib in combination with the DGK inhibitor, Amb639752. (D) Quantifications of GFP pixel intensity revealed that Amb639752, like Ritanserin, results in a synergistic reduction in tumour size together with Trametinib. Data from 1 experiment with 24 replicate wells for each treatment. (E) Binarized image of a drug test plate where larvae were treated with Trametinib in combination with the DGK specific inhibitor, CU-3. (F) Quantifications of GFP pixel intensity revealed that CU-3, like Ritanserin, results in a synergistic reduction in tumour size when combined with Trametinib. Data from 1 experiment with 24 replicate wells for each treatment. Statistical tests used were one-way ANOVAs with Tukey's multiple comparisons. Error bars represent S.E.M. ** = $p<0.01$, *** = $p<0.001$, **** = $p<0.0001$.

Disease Models & Mechanisms • Supplementary information

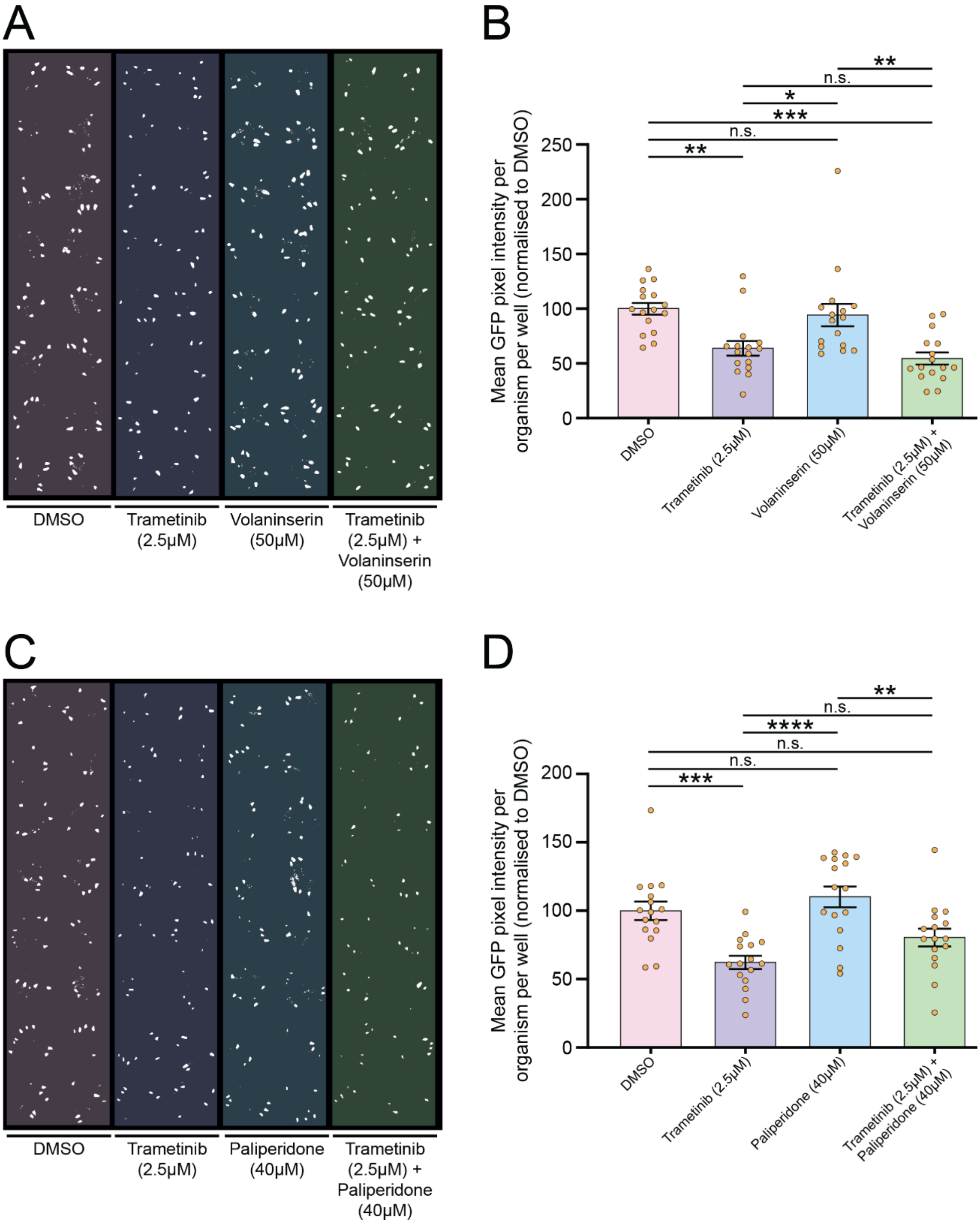

Fig. S7. Specific inhibition of serotonin receptors does not synergise with Trametinib to reduce *scrib⁻/Ras*^{V12} tumour size.

(A, B) Binarized image of a drug test plate where larvae were treated with Trametinib in combination with the 5HT inhibitors, Paliperidone (A) or Volaninserin (B). (C, D) Quantifications of GFP pixel intensity revealed that Paliperidone or Volaninserin do not synergise with Trametinib to reduce tumour size. Data from 1 experiment with 16 replicate wells for each treatment. Error bars represent S.E.M. A one-way ANOVA with a Tukey's multiple comparison test was used to measure statistical significance. * $p < 0.05$, ** $p < 0.01$, *** $p < 0.001$, **** $p < 0.0001$.

Disease Models & Mechanisms • Supplementary information

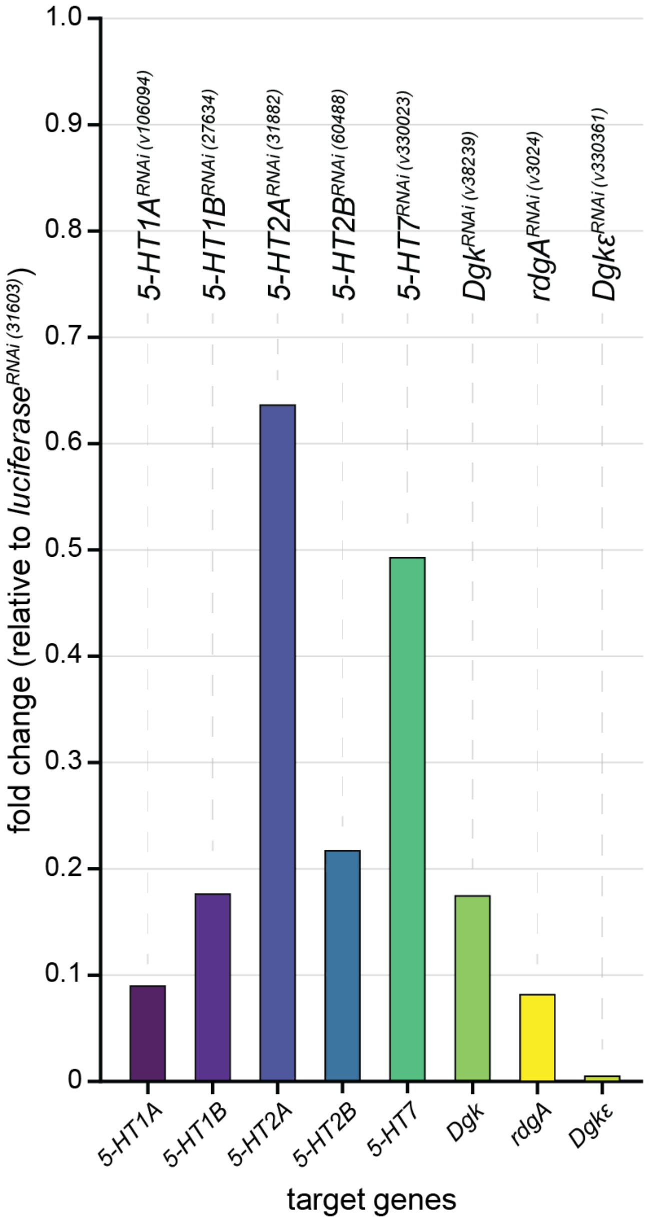

Fig. S8. RNAi lines utilised in this study are effective at reducing gene expression.

Quantification of qRT-PCR data testing the efficacy of the various *RNAi* lines utilised in Figure 4 relative to the *luciferase-RNAi* non-targeting control from mRNA extracted from pupal/adults expressing *Actin-GAL4* driven *UAS-RNAi* trangenes. Gene expression is displayed as fold change normalised to the non-targeting RNAi control. All *RNAi* lines employed were found to be effective at reducing gene expression.

Disease Models & Mechanisms • Supplementary information

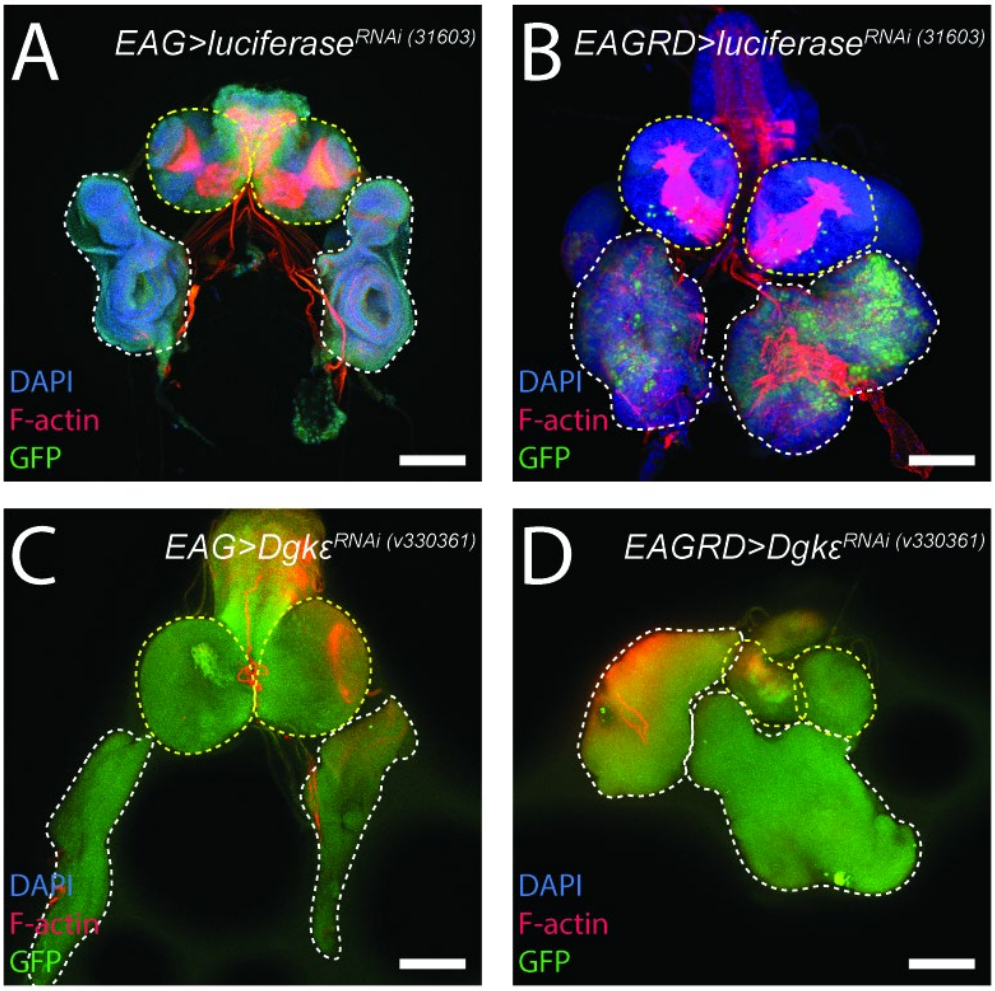

Fig. S9. Genetic analyses of *dlg1*^{RNAi}*/Ras*^{V12} tumours suggest *Dgkε* knockdown does not reduce tumour size.

(A-D) Brains from L3 animals with their attached *wild-type* (EAG>, A, C) or *dlg1*^{RNAi}*/ Ras*^{V12}-expressing (EAGRD>, B, D) eye-antennal discs, also expressing GFP (green) and RNAi against *luciferase* (as a control, A, B) or *Dgkε*, (C, D) stained with DAPI (blue, except for C, D) and for F-actin (red). As the DAPI staining failed for the samples with RNAi against *Dgkε*, we were unable to quantify the change in tumour size upon *Dgkε* knockdown. However, visually, it is clear that *Dgkε* knockdown had little to no effect on tumour size using this system. Brain lobes are outlined in yellow, and the eye-antennal discs are outlined in white. Scale bars represent 100μm.

Disease Models & Mechanisms • Supplementary information

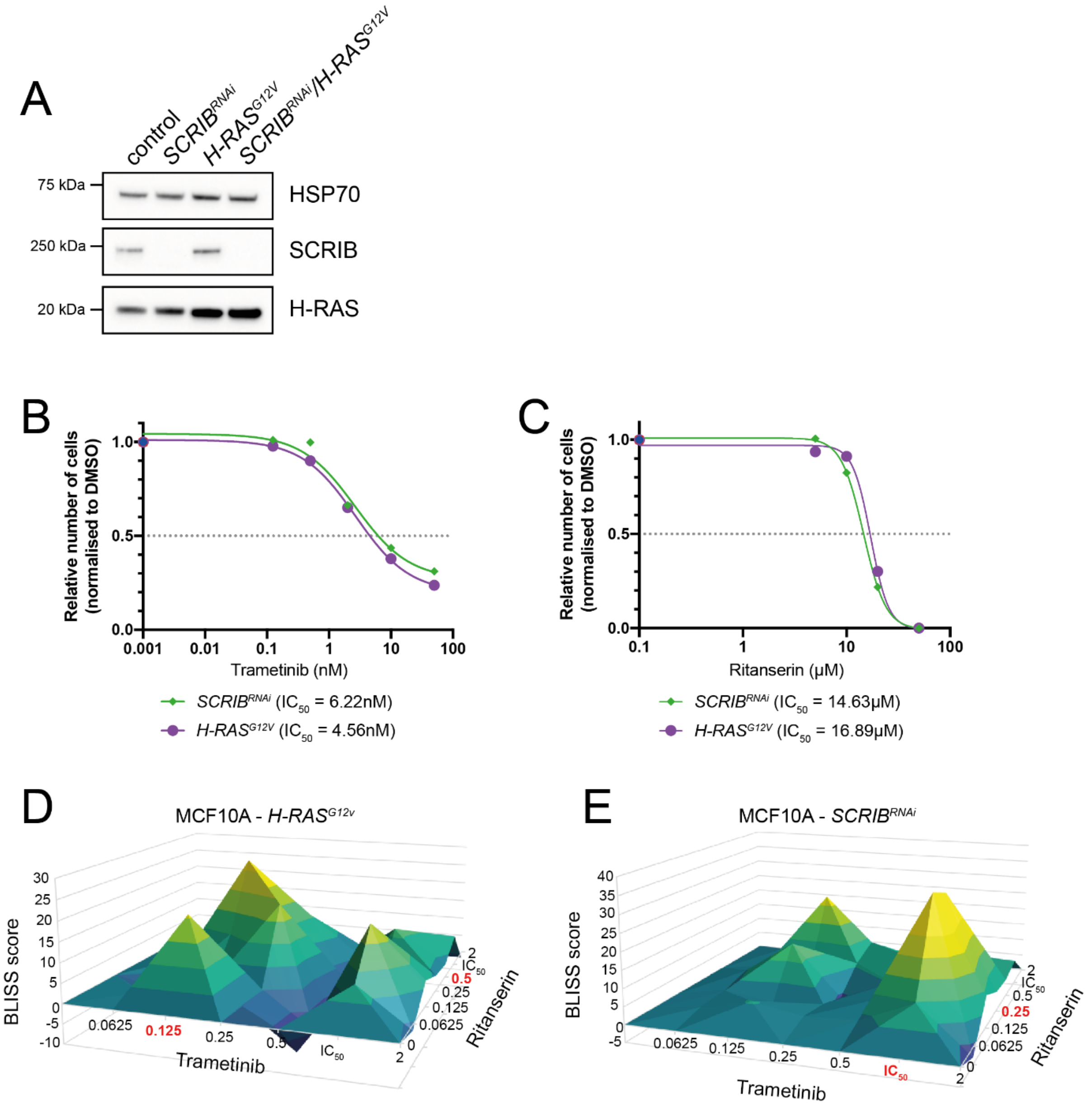

Fig. S10. Trametinib synergises with Ritanserin in cultured MCF10A cells separately expressing either *shRNA-SCRIB* or human *H-RAS^{G12V}*. (A) Western blotting confirmed *SCRIB* knockdown and *H-RAS^{G12V}* expression in MCF10A cells with the respective constructs. HSP70 was used as a loading control. (B,C) Dose response curves were generated for Trametinib (B) and Ritanserin (C) in MCF10A cells with knockdown of *SCRIB* or expression of *H-RAS^{G12V}*. Live cell proportions were determined via CellTiter-Glo assay, and IC_{50} values were calculated from 3 independent experiments. (D,E) BLISS synergy scores for *H-RAS^{G12V}* (D) and *shRNA-SCRIB* (E) MCF10A cells treated with Trametinib in combination with Ritanserin at the indicated doses. Doses approximate magnitudes of the IC50 for each drug. The proportion of live cells was determined by CellTiter-Glo assay, across 3 replicate experiments. A BLISS score > 0 indicates synergy.

Disease Models & Mechanisms • Supplementary information

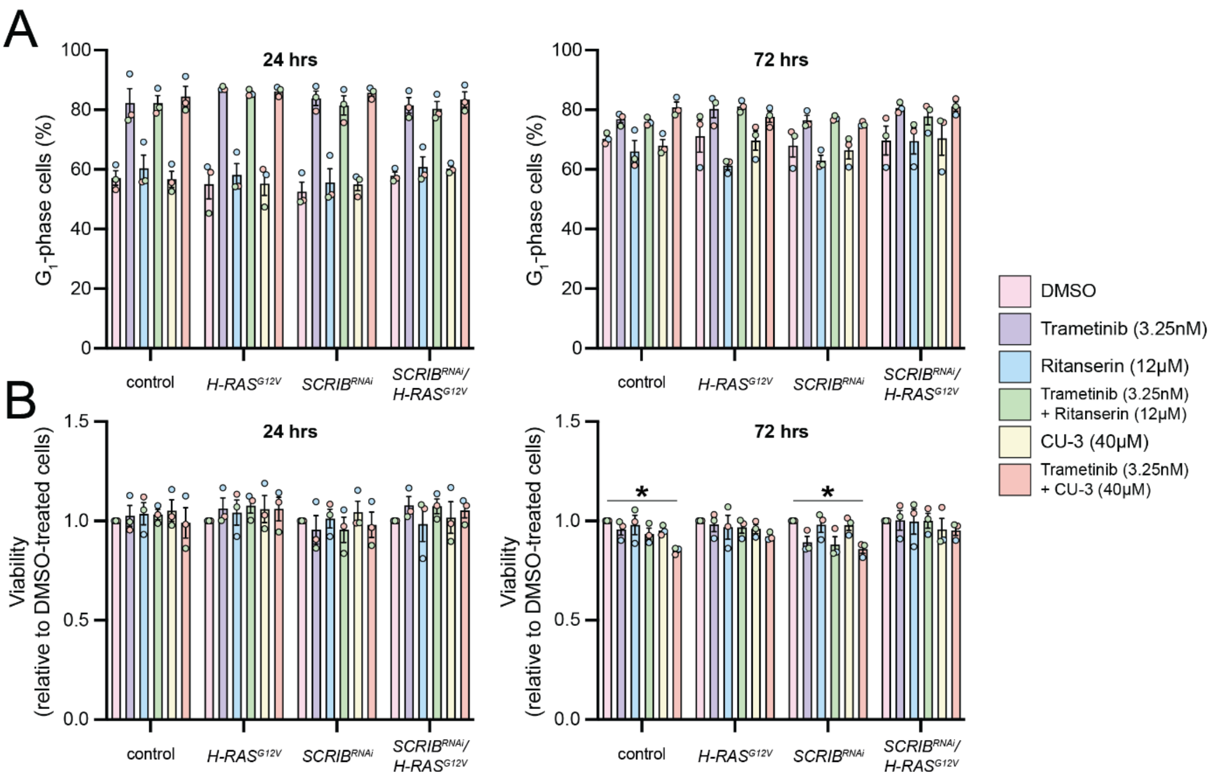

Fig. S11. Cell death and cell cycle assays of drug treated cells reveal that the cell cycle is arrested in G_1 phase by Trametinib treatment.

(A) The results of cell cycle assays conducted using control, *H-RAS*^{G12V}, *SCRIB*^{RNAi}, and *H-RAS*^{G12V}/*SCRIB*^{RNAi} MCF10A cells treated for 24 or 72 hrs with the various compounds-of-interest. Compounds were used at IC_{50} levels to ensure a clear readout of their effect on cell behaviour/survival was obtained. Overall, cells treated with Trametinib (either alone or alongside either Ritanserin or CU-3) possess a marked increase in G_1-phase cell numbers. This effect is more pronounced after 24 hrs of treatment and somewhat reduced after 72 hrs of treatment. (B) The results of viability assays conducted using control, *H-RAS*^{G12V}, *SCRIB*^{RNAi}, and *H-RAS*^{G12V}/*SCRIB*^{RNAi} MCF10A cells treated for 24 or 72 hrs with the various compounds-of-interest. Compounds were used at IC_{50} levels to ensure a clear readout of their effect on the cell cycle/survival was obtained. Minimal differences were observed between timepoints or treatments in the cell death assays, suggesting that the drugs largely do not drive apoptosis of these cells at 24 or 72 hrs. Separate experimental replicates are identified by different coloured circles. Error bars represent S.D. A one-way ANOVA with a Tukey's multiple comparison test was used to measure statistical significance within each cell line. * $p < 0.05$.

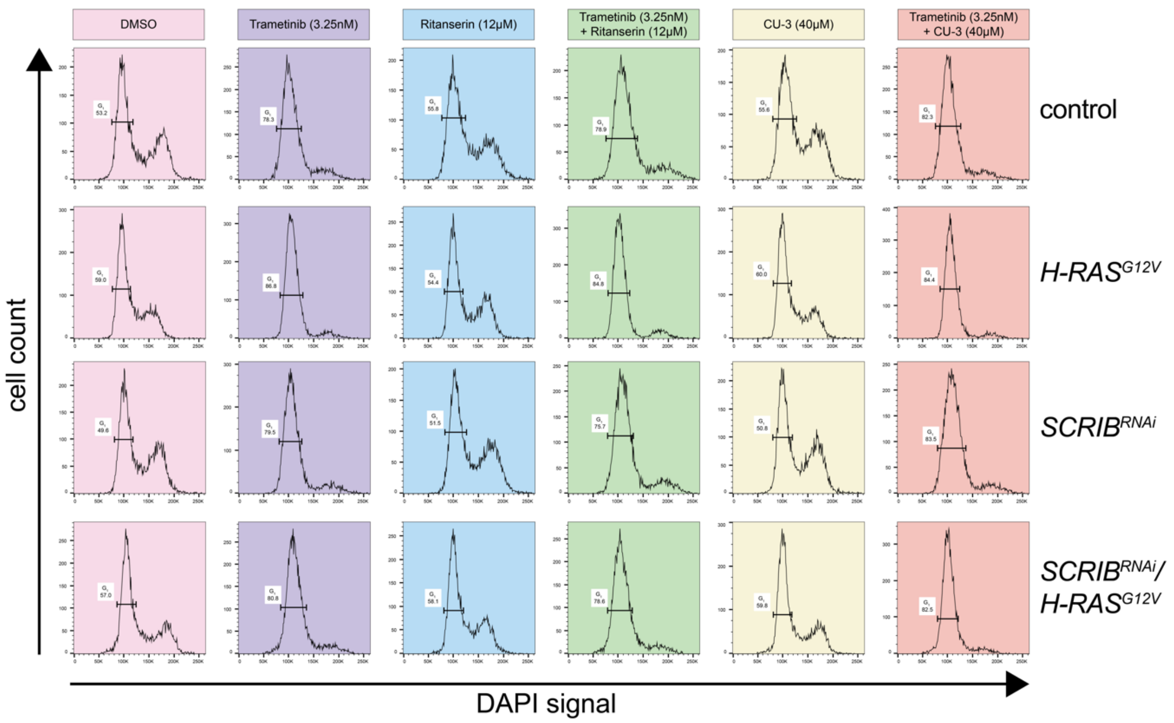

Fig. S12. Representative cell cycle FACS plots upon treatment of cells with Trametinib and DGKα drug combinations.

Representative FACS plots from assessments of the cell cycle for one of the 24 hr drug treatment replicates for control, *H-RAS*^{G12V}, *SCRIB*^{RNAi}, and *H-RAS*^{G12V}/*SCRIB*^{RNAi} MCF10A cells. Treatments are indicated along the top of the image, and are colour-coded. The y-axis indicates cell counts, and the x-axis indicates DAPI signal strength, which was used to assess cell cycle stages. Samples treated with Trametinib (either alone or with either Ritanserin or CU-3) possess a strong disruption to their cell cycle, with a clear increase in G_1-phase cell numbers, at the apparent expense of S- and G_2-phase cell numbers. However, the expansion of the G_1 peak in some samples made precise measurement of the proportions difficult – example assessments are shown on each plot. Each graph represents the results from >10,000 cells.

Disease Models & Mechanisms • Supplementary information

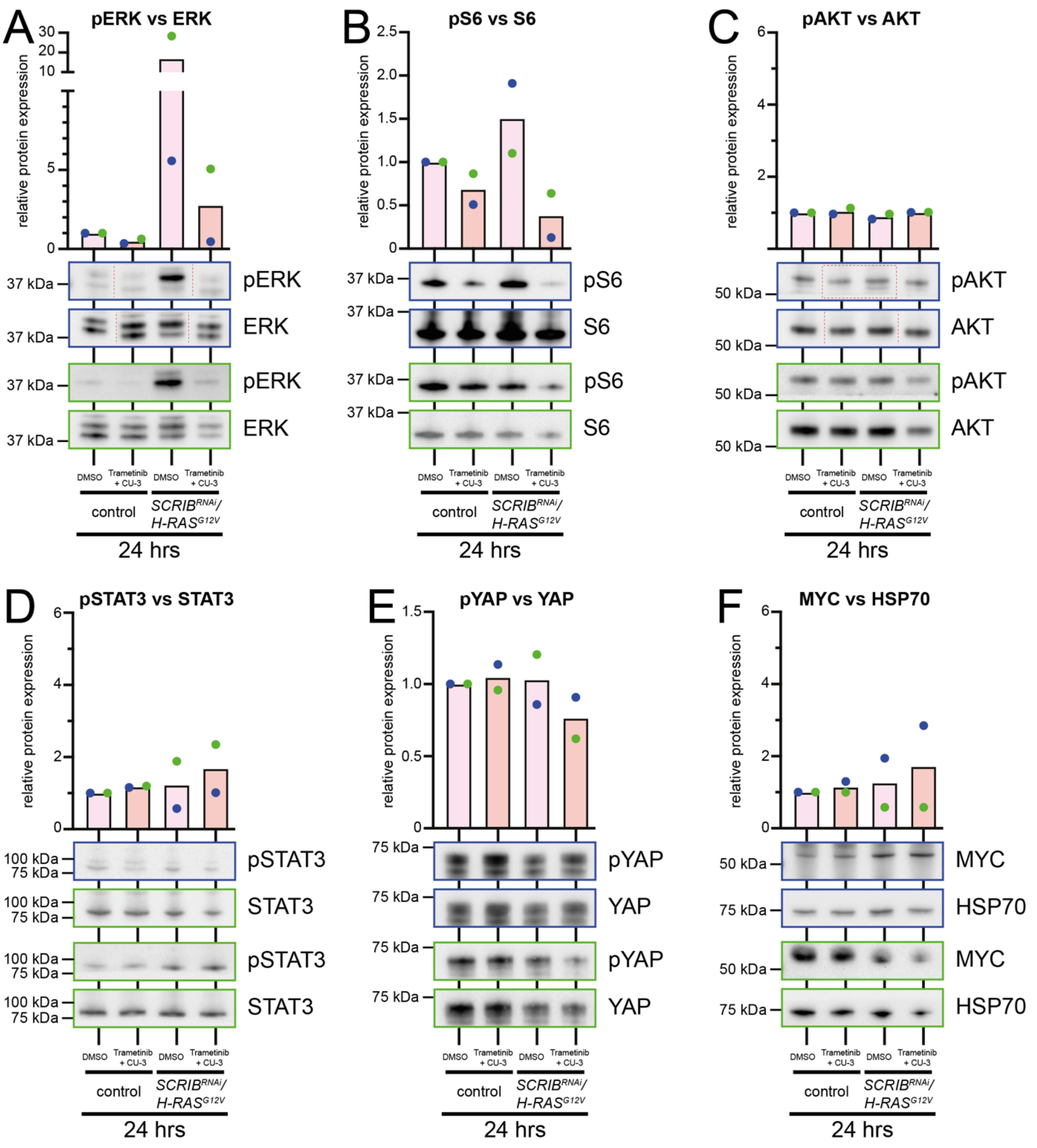

Fig. S13. Analysis of the effect on signalling pathways upon treatment with the DGKα inhibitor, CU-3, and Trametinib.

(A-F) Western blot images of MCF10A control or *SCRIB^{RNAi}/H-RAS^{G12V}* cells, treated for 24 hrs, either with DMSO or the highest synergy combination of Trametinib and CU-3. Each blot shown is from one of 2 independent replicates, the results of which are identified by colour-coded dots on each quantification. Quantifications are shown

Disease Models & Mechanisms • Supplementary information

normalized to the DMSO control samples. Our data reveals that in Trametinib and CU- 3 treated *SCRIB*^{RNAi}/*H-RAS*^{G12V} cells, relative to the DMSO control, there were strong reductions in pERK (A) and pS6 (B) levels. pAKT (C) levels were relatively unchanged as were pSTAT3 (D) levels, pYAP (E) levels and levels of the YAP target, MYC (F). Note, the order of some lanes in A and C were flipped to maintain a consistent loading order among all the images, as indicated by the dotted red outlines.

Disease Models & Mechanisms • Supplementary information

Table S1. Fly Stocks used in this study.
A list of stocks used throughout the project where the stock names indicate the genotype of the fly stock. VDRC is the Vienna *Drosophila* Resource Centre. BDSC is the Bloomington *Drosophila* Stock Centre.

Stock Name	Additional Names	Source	Catalogue Number	Additional Information
eyFLP, UAS-GFP ;; tub-GAL4, FRT82B, tub-GAL80 / TM6B-RFP	MARCM82B			
UAS-Ras85D^{V12} ; FRT82B, scrib^{1} / TM6B				
eyFLP ;; Act>>GAL4, UAS-GFP / TM6B	eyFLPout EAG	Louise Cheng		
eyFLP ; UAS-Ras85D^{V12}, UAS-dlg1 RNAi / CyO tub-GAL80 ; Act>>GAL4, UAS-GFP / TM6B	EAGRD	Konrad Basler		Willecke et al., 2011
hsFLP ; Act>>GAL4				
UAS-5-HT1A RNAi	*5-HT1A^{RNAi}*	VDRC	v106094	
UAS-5-HT1B RNAi	*5-HT1B^{RNAi}*	BDSC	27634	
UAS-5-HT2A RNAi	*5-HT2A^{RNAi}*	BDSC	31882	
UAS-5-HT2B RNAi	*5-HT2B^{RNAi}*	BDSC	60488	
UAS-5-HT7 RNAi	*5-HT7^{RNAi}*	BDSC	v330023	
UAS-Dgk RNAi	*Dgk^{RNAi}*	VDRC	v38239	
UAS-rdgA RNAi	*rdgA^{RNAi}*	VDRC	v3024	
UAS-Dgkε RNAi	*Dgkε^{RNAi}*	VDRC	v330361	
UAS-luciferase RNAi	*luciferase^{RNAi}*	BDSC	31603	Used as a non-targeting RNAi control
w^{1118}		BDSC	3605	Used as a wild-type

Disease Models & Mechanisms • Supplementary information

Table S2. Primers used in qRT-PCR analysis.

Gene	Forward Primer (5'-3')	Reverse Primer (5'-3')
Gapdh2	GCAAGCAAGCCGATAGATAAACA	CGTTGGCGCCCTTATCAATG
Dgk	CGGTGGCCATTCAGGACTTT	CGAAGTCCAGTCCTCACCTG
Dgkε	ATGGAGGTGTTCGGCATTGT	GGTCTCCTTGACTTGTAGCCTT
5-HT1A	TTGCCGTCGATCGTTACTGG	GCCGTCCAAACGCAAAAGAT
5-HT1B	GGAATCGCAGCATAAACGGC	ACGAATACGTTGCCTATGATGG
5-HT2A	CTTTTTCGCAGGTTGGGTGG	GTTGAAGTTGCGATTGCCGT
5-HT2B	GGAATAAAACACGTCGGCGG	CGAGGCGTGATTCTTGGAGT
5-HT7	CGAGAAGAAAGCCGATCCGA	GCAGTCGGTTTCGCAGACTA
rdgA	CATTCCATCGCCGGAGGTAA	GGAACATGTCCAGGCCCATT
Rpl32	CCAGTCGGATCGATATGCTAA	GTTCGATCCGTAACCGATGT

Table S3. Primary antibodies used in western blots.

Antigen	Host Species	Dilution	Source
pS6	Rb	1:2000	Cell Signaling Technology #4858
S6	Rb	1:10,000-100,000	Cell Signaling Technology #2217
pAkt	Rb	1:1000	Cell Signaling Technology #4060
Akt	Rb	1:1000	Cell Signaling Technology #4691
pERK	Ms	1:1000	Cell Signaling Technology #9106
ERK	Rb	1:500	Cell Signaling Technology #9102
pSTAT3	Rb	1:500	Cell Signaling Technology #9134/9145
STAT3	Rb	1:1000	Cell Signaling Technology #4904
pYAP	Rb	1:1000	Cell Signaling Technology #4911
YAP	Rb	1:1000	Cell Signaling Technology #14074
cMyc	Rb	1:1000	Cell Signaling Technology #5605
Hsp70	Ms	1:10,000	Lorraine O'Reilly and Robin Anderson
pP38	Rb	1:1000	Cell Signaling Technology #9211
P38	Rb	1:1000	Cell Signaling Technology #9212
H-RAS	Rb	1:1000	Sigma-Aldrich #04-775
SCRIB	Ms	1:1000	Santa Cruz Biotechnology #sc-55543

Disease Models & Mechanisms • Supplementary information

File S1. List of all drugs screened for synergy with Trametinib in reducing *scrib*⁻ *Ras*^{V12} tumour size.

Drugs highlighted in yellow are ones identified as reducing tumour size in the primary screen that were further explored in this study.

Click here to download File S1

File S2. Hits from the primary screen that did not significantly reduce *scrib*⁻ *Ras*^{V12} tumour size upon retesting.

Binarized images of the screening plates and graphs of pixel intensity are shown.

Click here to download File S2

File S3. Drug combination cell viability analysis.

Click here to download File S3

File S4. Code used to perform automated analyses of binarized images of 96 well plates containing larvae treated with drugs of interest.

Click here to download File S4
